# Short Carbon Fiber Reinforced Polymers: Utilizing Lignin to Engineer Potentially Sustainable Resource-Based Biocomposites

**DOI:** 10.3389/fchem.2019.00757

**Published:** 2019-11-08

**Authors:** László Szabó, Romain Milotskyi, Tetsuo Fujie, Takayuki Tsukegi, Naoki Wada, Kazuaki Ninomiya, Kenji Takahashi

**Affiliations:** ^1^Institute of Science and Engineering, Kanazawa University, Kanazawa, Japan; ^2^Innovative Composite Center, Kanazawa Institute of Technology, Hakusan, Japan; ^3^Institute for Frontier Science Initiative, Kanazawa University, Kanazawa, Japan

**Keywords:** lignin, biocomposite, carbon fiber, surface modification, discontinuous reinforcement, cellulose, polyamide 6

## Abstract

Carbon fiber reinforced composites have exceptional potential to play a key role in the materials world of our future. However, their success undoubtedly depends on the extent they can contribute to advance a global sustainability objective. Utilizing polymers in these composites that can be potentially derived from biomasses would be certainly vital for next-generation manufacturing practices. Nevertheless, deep understanding and tailoring fiber-matrix interactions are crucial issues in order to design carbon fiber reinforced sustainable resource-based biocomposites. In this study, cellulose derivatives (cellulose propionate and cellulose acetate butyrate) are utilized as model polymer matrices that can be potentially fabricated from biomasses, and the mechanical properties of the prepared short carbon fiber reinforced composites are engineered by means of a functional biobased lignin coating on the fiber surface. Furthermore, polyamide 6 based composites are also prepared, the monomer of this polymer could be obtained using C_6_ sugars derived from lignocellulosic biomasses in the future (through 5-hydroxymethylfurfural). Lignin was successfully immobilized on the carbon fiber surface via an industrially scalable benign epoxidation reaction. The surface modification had a beneficial impact on the mechanical properties of cellulose propionate and polyamide 6 composites. Furthermore, our results also revealed that cellulose-based matrices are highly sensitive to the presence of rigid fiber segments that restrict polymer chain movements and facilitate stress development. It follows that the physicochemical properties of the cellulosic matrices (molecular weight, crystallinity), associated with polymer chain mobility, might need to be carefully considered when designing these composites. At the same time, polyamide 6 showed excellent ability to accommodate short carbon fibers without leading to a largely brittle material, in this case, a maximum tensile strength of ~136 MPa was obtained at 20 wt% fiber loading. These results were further contrasted with that of a petroleum-based polypropylene matrix exhibiting inferior mechanical properties. Our study clearly indicates that carbon fiber reinforced polymers derived and designed using biomass-derived resources can be promising green materials for a sustainable future.

## Introduction

On account of a seemingly irreversible signature the human activity has already left on Earth, plastics among many manufactured modern materials can be found in geological deposits (Waters et al., 2016; Schneiderman and Hillmyer, 2017). It becomes, therefore, increasingly recognized that a new geological era, “Anthropocene” has certainly arrived due to the anthropogenic activity of the past decades (Waters et al., 2016). In light of the escalated situation, it is time to make right choices and consider the use of biobased biodegradable sources when designing materials (Chen and Patel, 2012; Sheldon, 2014; Graichen et al., 2017; Schneiderman and Hillmyer, 2017). As a result of tremendous research efforts, it appears that most of the important plastics can be derived from renewable sources (Chen and Patel, 2012; Schneiderman and Hillmyer, 2017), and these materials can be further tailored to make sustainable biocomposites for wide range of applications (Mohanty et al., 2018). In order to make the right decisions, deep understanding and tailoring the interactions between matrix segments in these biocomposites are essential, however, pose major scientific challenges in many cases.

Particular sustainability concerns are surrounding advanced structures that are recently gaining interest in several fields, and thereby are predicted to be key components in the materials world of the future. Based on their market growth and exceptional properties (Frank et al., 2014; Witten et al., 2017), carbon fiber reinforced polymers are these types of materials that must be strictly designed from the point of view of sustainability issues. In response to these concerns, pioneering work has started worldwide to prepare carbon fibers from renewable resources (Milbrandt and Booth, 2016). Among the possible candidates, lignin as the most abundant aromatic biopolymer has emerged as a very promising sustainable precursor, opening the gates for the production of low-cost biomass-derived carbon fibers (Fang et al., 2017). In respect to the polymer matrix of carbon fiber reinforced composites, intense research has been focused on changing the most frequently used thermoset epoxy matrix to recyclable thermoplastic resins targeting environmentally more benign compositions (Yao et al., 2018). Short carbon fiber reinforced thermoplastic composites are particularly attractive materials for industrial mass production due to their short production time and recyclability potential (Fu and Lauke, 1996; Fu et al., 2000; Karsli and Aytac, 2011, 2013; Ozkan et al., 2014; Unterweger et al., 2015).

Recent advances achieved in our laboratory hold promise for deriving thermoplastic cellulose derivatives from biomasses (Kakuchi et al., 2017; Suzuki et al., 2018), and utilizing these materials for preparing short carbon fiber reinforced plastics (Szabó et al., 2019a). We have also shown that the adhesion between the cellulosic matrix and carbon fiber can be improved by modifying the carbon fiber surface (Szabó et al., 2018a,b, 2019b). It appeared that immobilizing lignin on carbon fiber surface leads to a great enhancement in interfacial properties due to secondary interactions between lignin and the polymer matrix (Szabó et al., 2019b). It follows that lignin can serve as a functional material to improve the interfacial interactions in green biobased composites. In order to further test these theories, in this study we prepare short carbon fiber reinforced composites utilizing polymers that can be potentially derived from biomasses [cellulose derivatives (Kakuchi et al., 2017; Suzuki et al., 2018) and polyamide 6 (Buntara et al., 2011; Winnacker and Rieger, 2016)] as model materials and tailor the interfacial interactions utilizing lignin as a renewable resource.

While in our previous study we placed interfacial interactions under scrutiny (Szabó et al., 2019b), and therefore, surface modification was achieved via industrially and environmentally less attractive chemistries, here we bind lignin to the carbon fiber surface via a mild, environmentally benign and industrially scalable epoxidation reaction. The surface modified short fibers are embedded in the polymer matrices, and the macroscopic properties of the composites are extensively characterized. Our results are also compared to that obtained with a purely petroleum-derived polypropylene matrix.

## Experimental

### Materials

The short carbon fiber used in this study was a 3 mm long PX35 type fiber obtained from Zoltek (Toray Group, St. Charles, MO, USA). These chopped fibers have no sizing agent on the surface and are characterized by a nominal diameter of 7.2 μm. Prior to the experiments, the fibers were dried in a vacuum oven at 130°C for 72 h. In the cyclic voltammetry experiments due to the experimental setup, long fibers were used (T700SC-12000-50C, Toray Industries, Tokyo, Japan), in this case, the sizing agent was removed according to our previously elaborated procedure (Szabó et al., 2018a,b, 2019b).

As cellulose-based matrices, two commercially available thermoplastic esters (cellulose propionate and cellulose acetate butyrate) were used, these polymers showed excellent processability in preliminary experiments. Cellulose propionate (*M*_n_ ≈ 79 000 g mol^−1^) was purchased from Scientific Polymer Products, Inc. (Ontario, NY, USA; Catalog number 321), the degree of substitution (DS) value has been determined previously as DS = 2.76 (92% of the hydroxyl groups are functionalized) (Szabó et al., 2018a). Cellulose acetate butyrate (*M*_n_ ≈ 70 000 g mol^−1^) was provided by Eastman Chemical Company (Kingsport, TN, USA; Catalog number 381-20), the degree of substitution value is as follows: DS_acetyl_ = 1.00 (33.3% of the originally available hydroxyl groups), DS_butyryl_ = 1.66 (55.3% of the originally available hydroxyl groups). Furthermore, polyamide 6 resin (melt flow rate of 15 g per 10 min at 230°C) was obtained from Toray Industries (Tokyo, Japan; Catalog number CM1006). Polypropylene (melt flow rate of 25 g per 10 min at 230°C) was from Japan Polypropylene Corporation (Yokkaichi, Japan; Catalog number WINTEC™ WSX03). The cellulosic resins and polyamide 6 were dried in a vacuum oven at 120°C for 1 day before the experiments, and all the resins were kept in a dry box (containing silica gel) prior to use.

Kraft lignin (*M*_w_ ≈ 10000 g mol^−1^; low sulfonate content) was purchased from Sigma Aldrich (St. Louis, MO, USA; Lot # 04414PEV), distribution of the hydroxyl groups has been determined in our previous study as follows (Szabó et al., 2019b): OH_phenolic_ = 2.12 mmol g^−1^, OH_aliphatic_ = 1.65 mmol g^−1^, OH_carboxylic_ = 0.59 mmol g^−1^, OH_total_ = 4.36 mmol g^−1^.

Anhydrous pyridine, *N*,*N*-dimethylformamide (DMF) and ferrocenemethanol were provided by Sigma Aldrich (St. Louis, MO, USA). The solvents acetonitrile (ACN) and dichloromethane (DCM) were obtained from Naclai Tesque, Inc. (Kyoto, Japan) and Kanto Chemical (Tokyo, Japan), respectively. All the other chemicals were from Tokyo Chemical Industry Co., Ltd. (Tokyo, Japan).

### Synthetic Procedures

#### Lignin Modification

**Figure 1:**
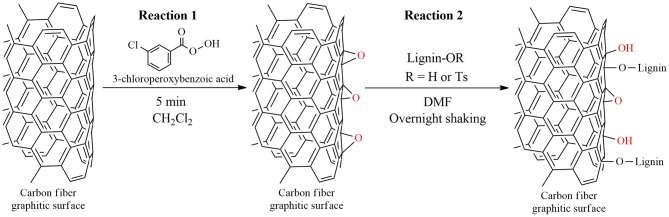
Representative reaction scheme depicting the functionalization procedure (illustrative example).

Kraft lignin was tosylated according to our previously reported procedure using tosyl chloride (and pyridine as solvent), a detailed description can be found elsewhere (Szabó et al., 2019b). The synthetic procedure affords tosylated lignin derivative with a tosyl group content of 0.26 mmol g^−1^ (calculated from elemental analysis). The tosylation procedure is necessary to render the original lignin soluble in the conventional solvent used for the grafting procedure (*N*,*N*-dimethylformamide, see Figure 1).

#### Carbon Fiber Surface Modification

The surface of carbon fibers was epoxidized according to a recently reported procedure applying mild conditions (Koutroumanis et al., 2018), the synthetic method was previously elaborated for carbon nanotubes (Ogrin et al., 2006). The grafting procedure is outlined in Figure 1.

Briefly, 6 g chopped carbon fibers are immersed in a solution containing 30 g 3-chloroperoxybenzoic acid [by dry weight, note that due to instability issues the reagent has around 30% water content; equals 29.2 mmol g^−1^ fibers, following the stoichiometries elaborated in our previous works (Szabó et al., 2018a,b, 2019b)] in 500 mL dichloromethane. The reaction is conducted for 5 min, and the fibers are immediately washed using around 1 L dichloromethane to remove the unreacted reagents, and then they are placed in a solution containing 10 g tosylated lignin in 300 mL DMF. The reaction is conducted at room temperature overnight in a double shaker operating at 80 min^−1^ speed (Double Shaker NR-30, Taitec Corporation, Koshigaya, Japan). The functionalized fibers are washed with water, dichloromethane and acetone. The fibers are then dried in a vacuum drying oven at 50°C for 24 h.

**Figure 2:**
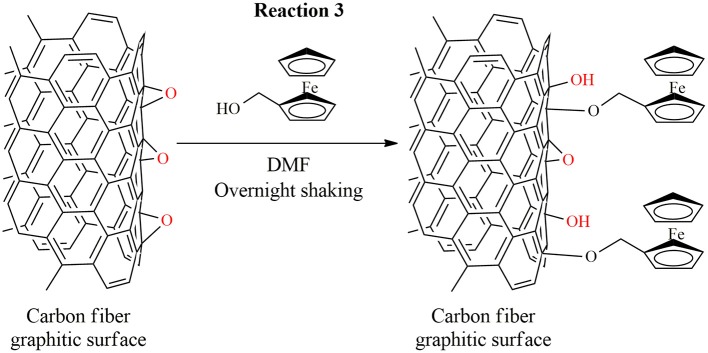
Representative reaction scheme for binding an electrochemical probe to the carbon fiber surface after the epoxidation step (illustrative example).

It should be noted that 5 min reaction time was reported to be sufficient to reach the maximum amount of epoxide functions on the surface (Koutroumanis et al., 2018). In line with these findings, we could not observe improvement in the amount of epoxide functions when we applied 10 min reaction time (electrochemical analysis after Reaction 3, see in Figure 2, note that data for 10 min are not shown).

### Cyclic Voltammetry Experiments

Cyclic voltammetry experiments were conducted to confirm the epoxidation reaction and prove the reactivity of the newly generated oxygen functions toward an aliphatic hydroxyl group. For this purpose, after the epoxidation reaction (Reaction 1 in Figure 1), 25 mg carbon fibers were placed in a solution containing 0.158 g ferrocenemethanol (0.73 mmol) in DMF, for 24 h at room temperature. After the reaction, the fibers were exhaustively washed according to the previously mentioned cleaning procedure, and then applied as working electrodes in the following cyclic voltammetry setup. The reaction is depicted in Figure 2.

The three-electrode cyclic voltammetry cell included a Pt auxiliary electrode, an Ag/Ag^+^ reference electrode (0.01 M AgNO_3_ and 0.1 M tetrabutylammonium perchlorate in acetonitrile; ALS Co., Ltd, Tokyo, Japan) and carbon fiber was applied as working electrode. The SVC3 type voltammetry cell was connected to an ALS/CH Instruments Electrochemical Analyzer Model 1200A potentiostat (ALS Co., Ltd, Tokyo, Japan). Further experimental details can be found in our previous work (Szabó et al., 2019b).

### Composite Preparation

Carbon fibers and the resin were compounded in an Xplore MC5 microcompounder equipped with short co-rotating twin-screw (Xplore Instruments BV, Sittard, The Netherlands). The composites were subjected to an injection molding step using an Imoto IMC-5705 injection molding machine to prepare standard dumbbell-shaped specimens (JIS K 7161-2:2014 1BA) for the mechanical tests. The experimental details for each type of resins are given in Table 1.

**Table 1:** Processing conditions for the extrusion and injection molding steps.

**Polymer resin**	**Extrusion process**			**Injection molding**		
	**Rotation speed**	**Retention time**	**Processing temperature**	**Barrel temperature**	**Mold temperature**	**Injection pressure**
Cellulose propionate	60 rpm	5 min	203°C	210°C	140°C	0.6 MPa
Cellulose acetate butyrate						
Polyamide 6			238°C	233°C	80°C	
Polypropylene			180°C	180°C	60°C	0.2 MPa

The flow characteristics of the resins were analyzed using a Shimadzu CFT-500EX Constant Test Force Extrusion Type Capillary Rheometer (Kyoto, Japan) following our previously reported procedures (Szabó et al., 2019a). The flow characteristics of the neat polymers can be found in the Supplementary Material (Figure S1).

### Mechanical Test

Tensile test was performed according to JIS K7161 standard (in line with ISO 527) using a Shimadzu Autograph AG-X Plus 5 kN tensile tester (Kyoto, Japan) equipped with a non-contact video type extensometer (TRViewX, Shimadzu; Kyoto, Japan). The crosshead-speed was 5 mm min^−1^ and a clamping pressure of 0.5 MPa was applied. Ten specimens were prepared for each composition.

Multiple *t*-test was performed to identify statistically significant differences (with a *P*-value < 0.05) in the results of the tensile test, assuming that the data represent a population with equal variance.

### Surface Analysis

X-ray photoelectron spectra were recorded on a Thermo Scientific K-Alpha X-ray Photoelectron Spectrometer System using Al K_α_ monochromated X-ray radiation (1486.6 eV, 36 W; Waltham, MA, USA). Data were collected from a spot size of 400 μm and the binding energy scale was calibrated to the hydrocarbon C1s peak at 285.0 eV. Survey spectra were obtained with a resolution of 1 eV and high-resolution C1s, N1s, O1s, and S2p spectra were recorded using a pass energy of 20 eV and a spectral resolution of 0.1 eV. Data were processed with a Thermo Scientific Avantage Software version 5.89 (Waltham, MA, USA). Overlapping peaks were resolved using the Powel method with Gauss-Lorentz Mix algorithm and a built-in Smart algorithm for background correction.

Morphological analysis was performed with a JSM-7610F field emission scanning electron microscope (FE-SEM) applying 8 mm working distance and 15 kV accelerating voltage (JEOL, Tokyo, Japan). Prior to the analysis, Au/Pd layer was sputtered on the sample surface using a Hitachi E1030 type equipment and a deposition time of 40 s (Hitachi, Ltd., Tokyo, Japan).

### Thermal Analysis

Thermogravimetric analysis was performed using a Shimadzu DTG-60AH thermal analyzer (Shimadzu Corporation, Kyoto, Japan) with a heating rate of 20°C min^−1^ under nitrogen atmosphere. Differential Scanning Calorimetry (DSC) experiments were conducted on a Shimadzu DSC-60A Plus equipment (Shimadzu Corporation, Kyoto, Japan) to determine the glass transition temperature of the polymers and their composites. DSC thermographs were recorded between −50 and 250°C using a heating rate of 10°C min^−1^ and a cooling rate of 50°C min^−1^. The second heating cycle was analyzed to determine the glass transition temperature.

### Fiber Length Analysis

Carbon fibers were extracted from the prepared composites using DMF as a solvent in case of the cellulosic matrices. Furthermore, for polyamide 6 and polypropylene based composites the matrix was separated from the fibers via a burning process performed at 550°C for 10 min in an electric furnace (ROP-001 type, ASONE Corporation, Osaka, Japan), and the obtained fibers were dispersed in water (Fu et al., 2000; Karsli and Aytac, 2013). Optical images of the fibers were recorded on an Olympus BX50 microscope (Tokyo, Japan). The images were analyzed with ImageJ program extended with the ridge/line detection algorithm (Steger, 1998).

## Results and Discussion

It has been revealed in our previous work that lignin can improve the shear tolerance at the carbon fiber-matrix interface in green cellulose-based (cellulose propionate) and commercially available epoxy-based composites (Szabó et al., 2019b). An exceptionally high interfacial shear strength was experienced as matrix failure took place before interfacial failure. Stimulated by the functional and green nature of lignin, we decided to take a step forward and scale-up the process using industrially attractive benign chemistries. Furthermore, we were not only interested in the macroscopic/mechanical outcome of the surface modification but also had a curiosity of how the interfacial effects are expressed for different type of matrices with sustainability potentials. Therefore, in addition to two cellulosic matrices (cellulose propionate and cellulose acetate butyrate), we extended our work to polyamide 6, which could be prepared using C_6_ sugars derived from lignocellulosic biomasses in the future (Buntara et al., 2011). Furthermore, we also compare these results with a potentially non-sustainable fully petroleum-based polypropylene matrix.

### Carbon Fiber Surface Modification

Our surface modification procedure was inspired by the convenient and efficient way of nanotube sidewall functionalization exploiting the ring-opening reactions between newly generated epoxide functions and nucleophilic agents (Markiewicz et al., 2014). Furthermore, the epoxidation process was recently applied for carbon fibers revealing the non-destructive, short, and benign nature of the functionalization procedure (Koutroumanis et al., 2018). Our surface treatment starts with a conventional epoxidation reaction carried out with short carbon fibers, which are placed in contact immediately with the tosylated lignin derivative (Figure 1). The main purpose of the tosylation reaction is to render the lignin derivative soluble in DMF. The tosylation procedure adopted from our previous study (Szabó et al., 2019b) affords a lignin derivative with a tosyl group content of 0.26 mmol g^−1^ (involving a range of aliphatic and aromatic hydroxyl groups, due to solubility issues ^31^P-NMR experiments could not be performed to investigate the hydroxyl group distribution profile), and leaves 4.1 mmol g^−1^ free hydroxyl groups behind. These free hydroxyl groups are expected to react with the epoxide groups present on carbon fiber surface. The nucleophilic attack on an epoxide typically follows S_N_2 mechanism, however, due to steric reasons (the reaction would require 180° attack on the epoxide carbon) this cannot be the case on the surface of carbon fibers. Nevertheless, it might be possible that the reaction eventually proceeds via a carbocation intermediate as it was shown before on arene oxides (Loew et al., 1980). Reactions on surfaces often follow atypical pathways owing to steric effects, the nucleophilic attack on the epoxidized carbon fiber surface might represent an interesting example, which would merit further attention.

**Figure 3:**
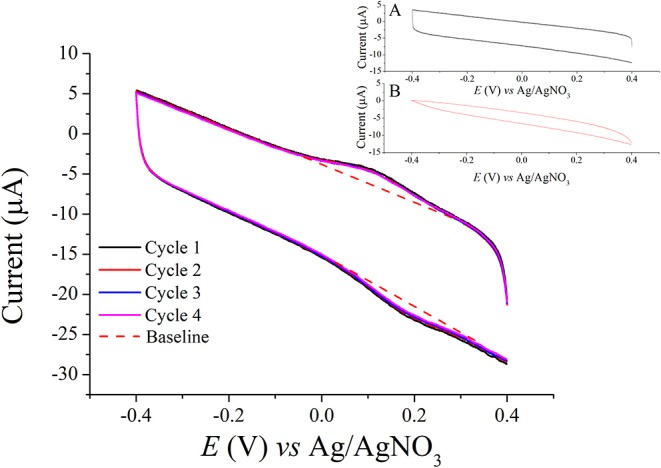
Cyclic voltammogram recorded with carbon fibers as working electrodes functionalized via Reaction 3 (Figure 2). Inset A shows the cyclic voltammetry response of a control sample; in this case Reaction 3 was performed with ferrocene instead of ferrocenemethanol to observe any interferences arising from π-π stacking interactions. Inset B shows another control sample, in this case unmodified carbon fibers (before epoxidation) were incubated with ferrocenemethanol under the conditions outlined in Reaction 3 to investigate the possibility of H-bonding interactions with surficial functional groups (-OH, -COOH, and -NH_2_ moieties).

The formation of epoxide groups, their reactivity as well as their quantity were investigated through cyclic voltammetry experiments. In these experiments, the epoxidized carbon fibers were reacted with ferrocenemethanol to immobilize the redox probe on the surface (Figure 2). Thereafter, the fibers were applied as working electrodes in a three-electrode cyclic voltammetry setup. The recorded voltammograms are shown in Figure 3. From the anodic and cathodic peaks, a standard electrode potential of *E*^0^ = 0.15 V vs. Ag/AgNO_3_ can be calculated, which is typical for ferrocene structures covalently bound to electrode surfaces (Umaña et al., 1980; Goff et al., 2010). The electrochemical response does not change over several cycles suggesting that stable structures have been immobilized on the substrates. Furthermore, in parallel experiments the possibility of π-π stacking interactions was firmly excluded since when ferrocene was applied in Reaction 3 (Figure 2) instead of ferrocenemethanol, no electrochemical response was obtained (Figure 3 Inset A). The possibility of H-bonding interactions between ferrocenemethanol and surficial functional groups [-OH, -COOH and -NH_2_ moieties that are known to be present on carbon fiber surface (Xie and Sherwood, 1989)] was also excluded as no electrochemical response can be seen for the corresponding control sample in Figure 3 (Inset B). By integrating the anodic/cathodic current (in Figure 3), the amount of transferred electrons can be calculated and thereby, the number of molecules determined. The surface coverage amounts to a value of 6.5 × 10^11^ molecules mg^−1^ according to these calculations. This value is much lower than that reported in our previous study (Szabó et al., 2019b), where we used much more aggressive chemistries (free radical-mediated reaction). However, it is considered that a high surface coverage might not be necessary for polymeric substrates having large amount of reactive functions in their structure. Therefore, the results of the cyclic voltammetry experiments indicate that the epoxidation reaction might be an appropriate measure to covalently bind lignin to the carbon fiber surface.

**Figure 4:**
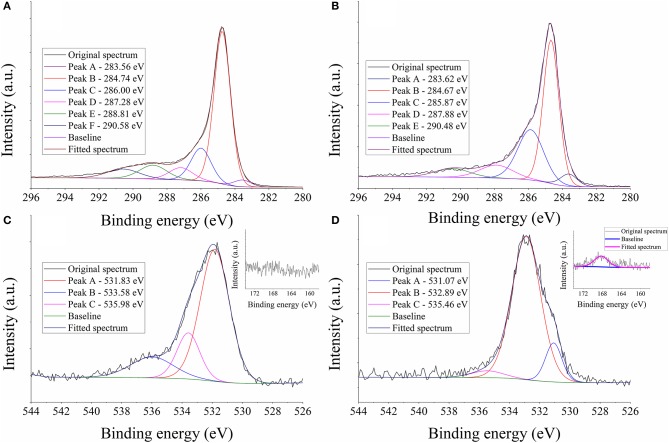
High-resolution C1s **(A,B)**, O1s **(C,D)** and S2p (Insets) X-ray photoelectron spectra of carbon fiber samples before modification **(A,C)** and after immobilizing lignin on the surface **(B,D)**.

In order to confirm the immobilization of lignin on the surface, the high-resolution C1s, O1s, and S2p X-ray photoelectron spectra of the unmodified and functionalized carbon fiber samples were recorded, the results are shown in Figure 4. The high-resolution C1s spectra of the unmodified/modified samples (Figures 4A,B) can be resolved into a main peak centered at ~285 eV, typical for the graphitic carbon present in the structure of carbon fibers (Xie and Sherwood, 1989; Zhang et al., 2008). The C1s spectrum of the unmodified sample (Figure 4A) consists of peaks typically assigned to C-OH/C-O-C (286.00 eV; note that C=N moieties also give signal around this region), C=O (287.28 eV) and –COOH (288.81 eV) groups (Xie and Sherwood, 1989). Furthermore, the peak located at higher energies (290.58 eV) is attributed to π-π^*^ shake up satellites (Xie and Sherwood, 1989; Zhang et al., 2008). In addition, the O1s spectrum (Figure 4C) is dominated by a peak centered at 531.83 eV, assigned to C=O or C-O-C moieties (Xie and Sherwood, 1989), while the peaks located at 533.58 and 535.98 eV are usually assigned to C=O groups and probably to some chemisorbed oxygen, respectively (Xie and Sherwood, 1989). After the functionalization process, an increase in the intensity of the peak located at ~286 eV is observable in the C1s spectrum (Figure 4B), this peak was assigned to –C-OH/C-O-C functions. Furthermore, the peak intensity at higher energies decreases, C=O and –COOH functions give signals around this region. The O1s spectrum also changes appreciably after the functionalization process (Figure 4D). It is dominated by a peak assigned to C-OH groups and the previously dominating peak arising from C=O signals is considerably suppressed. Therefore, the C1s and O1s spectra in line with each other indicate that the structure deposited onto the surface has large amount of –OH groups, typical for lignin. Furthermore, while the unmodified carbon fiber has no sulfur on its surface, after the functionalization process, sulfur can be clearly detected (Figures 4C,D, Insets). The broad feature of the peak that is observable in the S2p spectrum of the functionalized sample indicates that several types of sulfur-containing functions are present, which is reasonable since kraft lignin has sulfur in its structure originally, and sulfur is also incorporated in tosyl groups after the tosylation process. It should be pointed out that based on the XPS analysis, the lignin coated surface has free hydroxyl groups as well as tosyl functions. This is reasonable since the hydroxyl groups of lignin were only partially substituted as it was mentioned before. As a result of the XPS analysis, the chemical composition of the functionalized carbon fiber sample is in line with the structural features of a lignin derivative.

**Figure 5:**
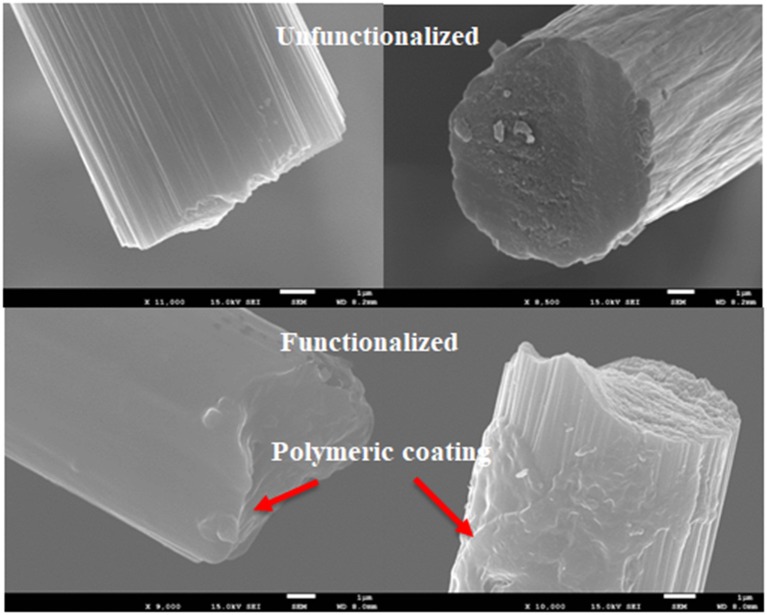
FE-SEM micrographs of carbon fiber samples before **(upper part)** and after **(lower part)** the functionalization process performed with the lignin derivative.

The FE-SEM micrograph of the unmodified sample shows the striations typical for unsized carbon fiber samples (Figure 5), while it is clear that a polymeric coating is present on the surface after the functionalization process. The deposited lignin coating composes a submicron layer on the surface.

We also performed thermogravimetric analysis (TGA) to provide more information about the amount of coating on the fibers, the results of the TGA analysis are shown in the Supplementary Material (Figure S2). Given that at 800°C around 44% weight loss takes place for the tosylated lignin sample, and assuming that the same process occurs at the carbon fiber surface, we can predict that around 3% lignin is present as coating on the surface by weight. The amount of lignin coating on the carbon fiber could be controlled by decreasing the amount of epoxide functions on the surface (e.g., by changing the reaction time, note that the amount of epoxide functions on the surface reaches a plateau in our study), this issue will be the matter of future optimization experiments.

In the following sections, short carbon fiber reinforced composites will be prepared using the surface modified carbon fibers (along with unmodified control samples), and their mechanical properties will be evaluated in light of their morphological features, fiber-length distribution and fiber orientation in the composite.

### Effect of Surface Modification on the Mechanical and Morphological Properties of Short Carbon Fiber Reinforced Composites

The mechanical properties of a short fiber reinforced composite can be discussed in light of a range of interconnected phenomena involving interfacial interactions, fiber length, and orientation distribution factors, the latter two being extremely sensitive to processing conditions (Fu and Lauke, 1996; Molnár et al., 1999; Fu et al., 2000; Karsli and Aytac, 2011, 2013; Ozkan et al., 2014; Li et al., 2015; Unterweger et al., 2015). The mechanical properties (σ_cu_–the strength of the composite) can be predicted using the frequently referred modified rule of mixtures theory as it is outlined in Equation 1 (Fu and Lauke, 1996; Fu et al., 2000; Ozkan et al., 2014; Li et al., 2015):

(1) $$\begin{array}{l} \sigma_{\text{cu}}=\chi_{1}\chi_{2}V_{f}\sigma l+V_{m}\sigma m \end{array}$$

where σ_l_ and σ_m_ are the strength, *V*_f_ and *V*_m_ are the volume fraction of the fiber and matrix, respectively. χ_1_ and χ_2_ are the fiber orientation and fiber length factors, respectively. In this expression (Equation 1), the interfacial interactions are included in χ_2_ as it involves the critical fiber length (*l*_c_) given by Equation 2 according to the Kelly-Tyson model (Kelly and Tyson, 1965):

(2) $$\begin{array}{l} l_{c}=\;\frac{\sigma_{fu}r}{\tau } \end{array}$$

where σ_fu_ is fiber tensile strength at the critical length [determined via single fiber tensile strength experiments (Kettle et al., 1997)], *r* is the fiber radius and τ is the interfacial shear strength characterizing fiber-matrix interactions.

According to these theories, the results of the mechanical tests will be discussed in terms of interfacial interactions [morphology of fracture surfaces (see Figures S3–10)], fiber orientation, and fiber length distribution aspects (see Figures S11–14). It has been shown in our previous work that lignin can improve interfacial adhesion due to secondary interactions and probably also due to plasticizing effects, and thereby it can serve as a functional material at the interface (Szabó et al., 2019b). Interfacial mechanical properties will not be further determined for the different matrices herein (it will be followed by examining morphological changes on the fracture surfaces). Whether the beneficial interfacial effects previously discovered can be transferred to the macroscopical properties of different composites will be the focus of the following sections.

#### Composites Based on Cellulosic Matrices

**Figure 6:**
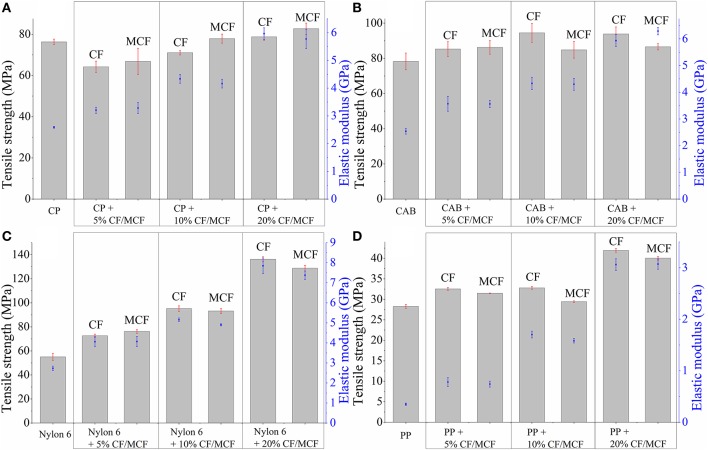
Tensile strength and elastic modulus values for **(A)** cellulose propionate (CP), **(B)** cellulose acetate butyrate (CAB), **(C)** polyamide 6 (Nylon 6), and **(D)** polypropylene (PP) based composites. CF, carbon fiber; MCF, modified carbon fiber (lignin coated).

**Figure 7:**
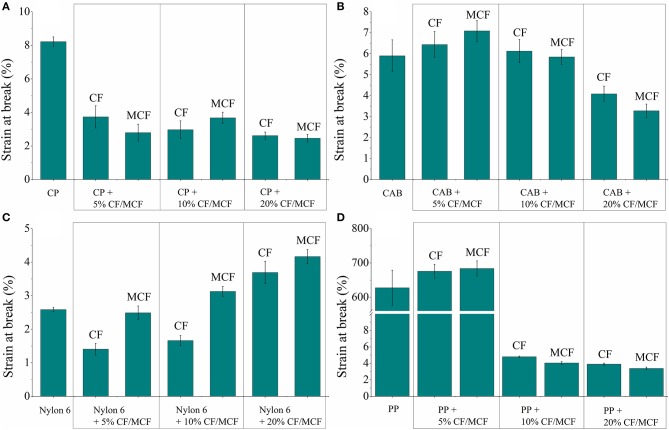
Strain at break values for **(A)** cellulose propionate (CP), **(B)** cellulose acetate butyrate (CAB), **(C)** polyamide 6 (Nylon 6), and **(D)** polypropylene (PP) based composites. CF, carbon fiber; MCF, modified carbon fiber (lignin coated).

**Figure 8:**
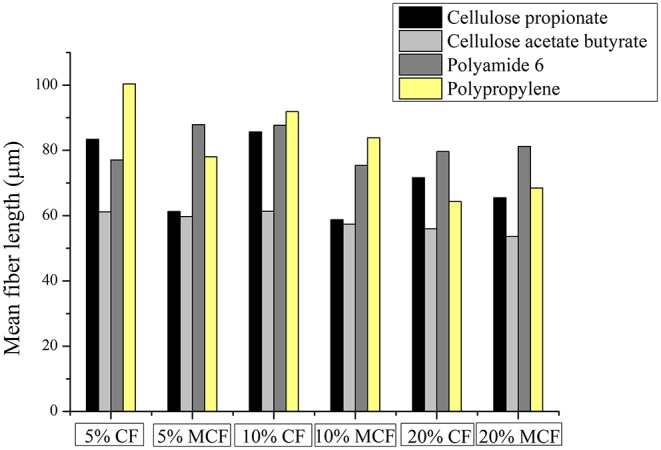
Mean fiber length in the composite samples. CF, carbon fiber; MCF, modified carbon fiber (lignin coated).

The tensile test results of the composite samples prepared using cellulose propionate (CP) and cellulose acetate butyrate (CAB) matrices are shown in Figures 6A,B, respectively (strain at break values are shown in Figures 7A,B). The tensile strength of the neat polymers lies very close to each other (~80 MPa, see Figures 6A,B). Nevertheless, CP and CAB show quite different response for the presence of fibers. The tensile strength decreases initially when 5 wt% short carbon fiber is present in the CP matrix (Figure 6A), however, an increase in tensile strength can be observed for the CAB matrix. It has been observed in previous studies that the presence of fibers in a composite can bring about an increase but also a decrease in composite strength (Fu et al., 2000). Due to the shear that develops during the extrusion and injection molding steps when preparing the composites, the initial fiber length (3 mm) decreases appreciably (in our study the mean fiber length is 50–100 μm (Figure 8), similar results can be found in other studies (Molnár et al., 1999; Karsli and Aytac, 2013; Ozkan et al., 2014; Li et al., 2015). When the fiber length drops below the critical fiber length determined by interfacial interactions, the fibers do not exhibit considerable reinforcing effects, however, their mere presence can diminish mechanical properties since they can serve as cores for crack formation and propagation leading to material failure (Fu and Lauke, 1996; Molnár et al., 1999). The fiber length observed in our study is much lower than the critical fiber length determined in our previous work for the cellulose propionate matrix (~840 μm) (Szabó et al., 2018a,b), which makes it possible that no reinforcement occurs at low fiber content in the CP matrix. At the same time, it is not expected that the critical fiber length differs considerably for the CAB matrix. Therefore, it is surprising that in this case an increase can be observed in tensile strength as 5 wt% fiber is included in the composite (Figure 6B). The difference between the CP and CAB matrices can possibly be connected to the variations in the freedom of polymer chain movements in the prepared composites. The addition of rigid fiber segments to the composite restricts the movement of molecular chains and increases the possibility of microcrack formation (Karsli and Aytac, 2011). Since the molecular weight of CAB (*M*_n_ ≈ 70 000 g mol^−1^) is smaller compared to CP (*M*_n_ ≈ 79 000 g mol^−1^), the mobility of polymer chains might be higher for CAB. This might be associated with a smaller extent of stress development and accumulation when rigid segments are present in the CAB matrix, giving an explanation for our findings. It should be noted, however, that increase in crystalline regions can also be connected to more restricted polymer chain mobility (this is not further evaluated herein).

At the same time, a monotonous increase in elastic modulus is observed for both the CP and CAB matrices with increasing fiber content, as a stiffer material is formed. It is known that the elastic modulus is mostly dependent on the fiber volume fraction and therefore, no strict correlation can be found with fiber length or surface modification in our study either (Figures 6A,B) (Molnár et al., 1999; Fu et al., 2000).

**Figure 9:**
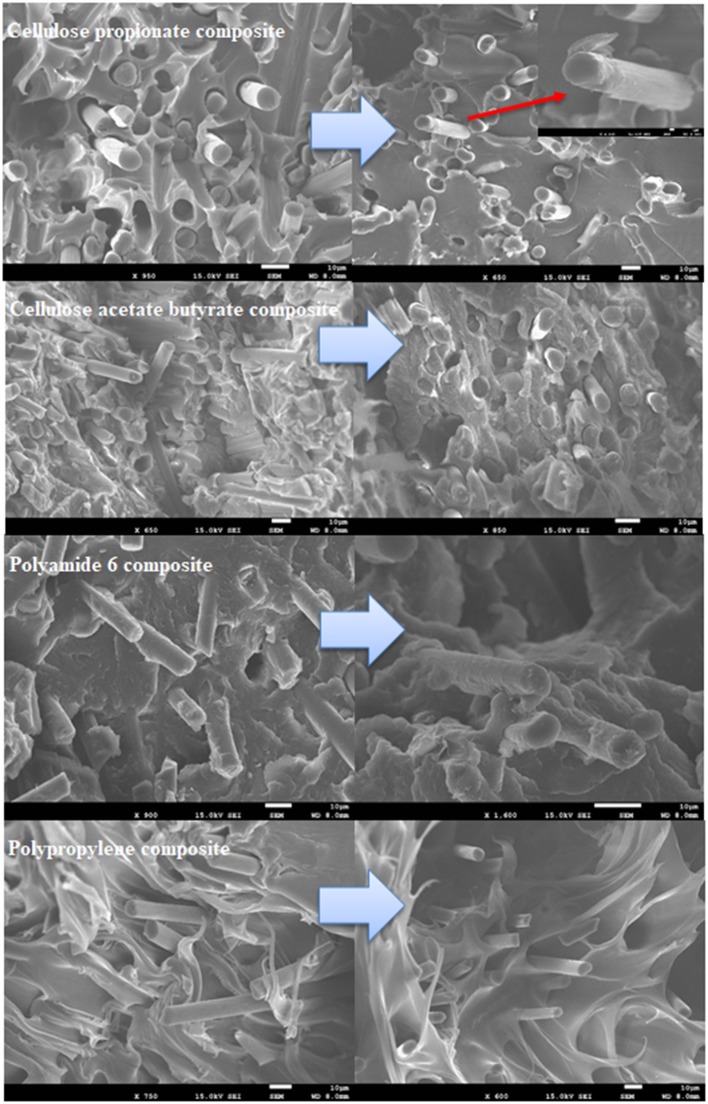
Fracture surfaces of composite samples containing 20 wt% carbon fiber. Pictures on the left and right site depict composite samples prepared with unmodified and modified carbon fibers, respectively.

The initial decrease in tensile strength is followed by a monotonous increase as the fiber content increases in the cellulose propionate matrix (Figure 6A). The tensile strength further increases when lignin is immobilized on the surface (at 20 wt% carbon fiber loading ~8% improvement in tensile strength could be obtained compared to the neat polymer matrix). Since the presence of lignin increases fiber-matrix adhesion for the cellulose propionate matrix (Szabó et al., 2019b), the load that is applied during tensile test is more efficiently transferred to the inherently stronger fiber, eventually leading to a higher tensile strength. This improved fiber-matrix adhesion can clearly be noticed on the fracture surfaces (Figure 9, Cellulose propionate composite) since after the functionalization process, polymer is attached to the fiber surface and also tight connection is observable between the matrix and fibers compared to the composite containing unfunctionalized fibers. Furthermore, as fibers are included in the matrix the strain at break value decreases to its half, exhibiting no significant dependence on fiber volume fraction or surface modification (Figure 7A). The decrease in strain at break is explained by the presence of the rigid filler that restricts polymer chain mobility and thus facilitates microcrack formation (Karsli and Aytac, 2011). Furthermore, it is also known that on account of the stress accumulation at the fiber ends, strain at break value decreases with increasing number of fiber ends (Fu et al., 2000; Karsli and Aytac, 2011, 2013). In our case, it appears that the brittleness of the material already reaches its limit after adding 5 wt% carbon fiber in the composite (i.e., no further decrease in strain at break can be observed). The mean fiber length does not show appreciable variation with increasing fiber content for the CP matrix (Figure 8; fiber length distributions can be found in Figure S11). Interestingly, when lignin is present on the surface, the fiber length appreciably decreases. This phenomenon can be explained in terms of the enhanced fiber-matrix interaction that results in the development of increased shear stress during the composite preparation.

An increase in tensile strength can be noticed for the CAB matrix with increasing unmodified fiber content (Figure 6B). The tensile strength, however, decreases when surface modified fibers are added to the matrix, indicating that lignin coating results in disadvantageous fiber-matrix interactions. In line with this phenomenon, the fracture surfaces do not indicate improved matrix adhesion to the fibers (Figure 9), and also the mean fiber length remains relatively constant with increasing fiber content (Figure 8; fiber length distributions can be found in Figure S12) anticipating poor fiber-matrix interactions. This poor interaction might be attributed to the presence of longer butyryl chains compared to the propionyl groups in CP (note that the degree of substitution value is DS_butyryl_ = 1.66 and DS_acetyl_ = 1.00 for CAB and DS_propionyl_ = 2.76 for CP), which might restrict secondary interactions (steric reasons or due to enhanced hydrophobicity of CAB) between lignin and the –OH and C=O groups of the cellulose derivative. Furthermore, the strain at break value does not change appreciably with increasing fiber content until 10 wt% fiber loading, and it drops to its half when 20 wt% fibers are present in the composite. This drop can be associated with a thicker skin layer in this type of composite containing fibers perpendicularly aligned to the flow direction (see Figure S6), this alignment can also be attributed to weak polymer-fiber interactions. At 10 wt% carbon fiber content (unmodified fiber surface) a maximum tensile strength of ~94 MPa is obtained (~20% improvement compared to the neat polymer matrix, Figure 7B), while the brittleness of the material does not change considerably if compared with the neat polymer (see the strain at break values in Figure 7B). This type of composite has therefore, superior properties over any other cellulose-based short carbon fiber reinforced polymers prepared in this study.

#### Composites Based on Polyamide 6

The tensile test results for the composites prepared using polyamide 6 as matrix are shown in Figures 6C, 7C. The tensile strength and modulus significantly increase as fibers are added to this polymer, and it appears that surface modification has no impact on these properties. A large increase in tensile strength is obtained at 20 wt% fiber loading, with exceptional ~250% improvement (~136 MPa; Figure 6C, unmodified fibers) compared to the neat polymer. This large enhancement can be attributed to very good fiber-matrix adhesion, as it can be seen on the fracture surfaces in Figure 9. Both the functionalized and unfunctionalized fibers are well-covered by the polymer, the interfacial layer resists the applied load during the tensile test, and it appears that matrix failure occurs first. The fiber orientation at the skin and at the core of the specimen is parallel to the flow direction (see Figures S7, S8), which is also advantageous with respect to the mechanical properties. In this work, the unmodified short fibers have no sizing agent on the surface, and it appears that this results in beneficial fiber-polymer interactions. In a previous study, short fibers with sizing agent have been applied, in that case, on the fracture surfaces the fibers are well-separated from the matrix and also less improvement could be obtained in the tensile strength results compared to our findings (at 20 wt% carbon fiber loading a tensile strength of ~90 MPa has been reported) (Karsli and Aytac, 2013).

The fiber length mostly remains longer in polyamide 6 based composites compared to the cellulosic matrices (Figure 8). Furthermore, an interesting phenomenon can be noticed on the strain at break values, as the carbon fiber surface is functionalized with lignin the fracture strain increases (Figure 7C). Such an increase might be connected to plasticizing effects acting at the interface, which can help in dissipating the stress along the fiber axis, eventually rendering the material less prone to stress accumulation. When 20 wt% carbon fiber is embedded in the matrix, the strain at break value is appreciably improved regardless of the surface modification. This might be attributed to fiber-fiber interactions [“bridge effect” (Fu et al., 2000)] due to the high fiber loading, helping to dissipate the stress.

It is noteworthy to mention that the polyamide based composite containing 20 wt% carbon fiber has superior characteristics in terms of tensile strength and fracture strain.

#### Composites Based on Polypropylene

It is obvious on the tensile test results that short carbon fiber reinforced polypropylene composites have inferior mechanical properties compared to other composites prepared in this study (Figure 6D). The tensile strength is slightly increased at 5 and 10 wt% fiber loadings (~14% increase compared to the neat polymer), and it reaches ~40 MPa at 20 wt% fiber content (~50% increase compared to the neat polymer). The large increase at relatively high fiber loading is attributed to fiber-fiber interactions resulting in “bridge effects” (Fu et al., 2000). It appears that surface modification detrimentally affects the mechanical properties as slight decrease in tensile strength can be noticed. It is reasonable to think that the originally hydrophilic lignin with -OH and -COOH groups in its structure will not establish beneficial secondary interactions with the polypropylene matrix that has a hydrophobic character. On the fracture surfaces, fiber-matrix separation can be observed, which becomes more significant after surface modification (Figure 9). The compatibility between polypropylene and lignin can, however, be improved if lignin is functionalized with aliphatic groups, as it has been shown in other studies from our laboratory (Sakai et al., 2018a,b). The elastic modulus monotonously increases (Figure 6D) with fiber loading, this is in line with previous reports pointing out that this mechanical property is mainly dependent on the fiber volume fraction and less dependent on other factors (Molnár et al., 1999; Fu et al., 2000). The mean fiber length remains relatively longer in these composites compared to other cases due to poor fiber-matrix interactions (Figure 8). Furthermore, while the material retains its ductile nature at 5 wt% fiber loading, the fracture strain falls to a low value after 10 wt% fiber is included in the polymer matrix (Figure 7D).

### Thermal Analysis

It has been shown before that the presence of fibers has an effect on the glass transition temperature (*T*_g_) of a polymer (Rezaei et al., 2009; Karsli and Aytac, 2013). At low carbon fiber loading, it was reported that fibers can increase the degree of crystallinity of the polymer matrix via increasing crystal growth rate and the number of nucleation sites, however, they can exert opposite effects as the amount of fiber increases in the matrix (Karsli and Aytac, 2013). Furthermore, it has also been shown that glass transition temperature increases with increasing fiber length (Rezaei et al., 2009). It is clear from these studies that glass transition temperature of short carbon fiber reinforced composites stems from the complex interplay of many factors.

The glass transition temperature values were determined for the cellulosic matrices and for polyamide 6 (the polypropylene used in this study is a metallocene catalyzed random copolymer, which made it difficult to analyze the wide range of glass transitions appearing during the thermal analysis), including the composites with 20 wt% fiber loading (the highest fiber loading was chosen since the amount of sizing on the surface is only ~3 wt%). As it can be seen in Table 2, the *T*_g_ value is not affected by the presence of fibers appreciably in case of the cellulosic matrices, however, a slight decrease occurs when lignin is present on the surface. The *T*_g_ value decreases more significantly for polyamide 6 after adding fibers, and an even sharper drop is observable when lignin-coated fibers are applied. The similar tendency that can be seen for these polymer matrices indicates that similar phenomenon might take place that eventually determines the glass transition in these materials when lignin is present at the interface. The mobility of polymer chains might increase close to the interface where lignin is present compared to the case when polymer chains face a rigid graphitic carbon layer at the interphase. When we compare the results of the mechanical tests and the thermomechanical analysis, it becomes clear that we cannot draw conclusion connecting the glass transition temperature and the mechanical properties of the materials. This might be due to the fact that these mechanical properties are determined mostly by the bulk matrix properties for short fiber reinforced composites and interfacial interactions (the interphase represents only a small fraction of the matrix) might be of lesser importance.

**Table 2:** Glass transition temperature values.

	**Glass transition temperature (°C)**
CP	129.8
CP + 20% CF	129.5
CP + 20% MCF	128.9
CAB	132.3
CAB + 20% CF	132.2
CAB + 20% MCF	131.6
Nylon	54.8
Nylon + 20% CF	53.5
Nylon + 20% MCF	51.4

*CP, cellulose propionate; CAB, cellulose acetate butyrate; CF, carbon fiber; MCF, modified (lignin coated) carbon fiber*.

## Conclusions

Our study aimed at preparing short carbon fiber reinforced composites using polymers that can be potentially derived from biomasses (cellulose derivatives, and polyamide 6) and thereby, have promising sustainability potential. Furthermore, interfacial interactions were engineered by immobilizing lignin, a renewable resource, on carbon fiber surface applying industrially scalable benign chemistries.

It appears that cellulosic matrices are relatively sensitive to the presence of rigid fiber segments that restrict polymer chain movement and facilitate microcrack formation. Increasing fiber-matrix interactions appears to bring about only slight improvement in the mechanical properties. Therefore, in these cases, further advancement might be achieved considering the physicochemical properties of the polymers (molecular weight, crystallinity).

At the same time, polyamide 6 has exceptional potential to accommodate fibers. A large improvement in tensile strength was obtained (reaching a maximum value of ~136 MPa at 20 wt% fiber loading) without leading to a largely brittle material.

Our study makes it clear that biomass-based short carbon fiber reinforced polymers can be promising green materials for a sustainable resource based society.

## Data Availability Statement

All datasets generated for this study are included in the article/Supplementary Material.

## Author Contributions

All the authors have contributed to the work presented in the manuscript to an extent that is consistent with the criteria for authorship. All the authors have agreed with the contents.

### Conflict of Interest

The authors declare that the research was conducted in the absence of any commercial or financial relationships that could be construed as a potential conflict of interest.
