# Supplementary material for: Short Carbon Fiber Reinforced Polymers: Utilizing Lignin to Engineer Potentially Sustainable Resource-Based Biocomposites
Source: Front Chem. 2019 Nov 8;7:757. doi: 10.3389/fchem.2019.00757 (PMC6857619; doi:10.3389/fchem.2019.00757)
Supplement: Supplementary file 1 [file Table_1.docx]

Supplementary Material

1. **Flow characteristics of the polymer resins**

**Figure S1.** Flow characteristics of the polymer resins. *T*_s_ – softening temperature, *T*_fb_ – flow beginning temperature.

1. **Thermogravimetric analysis**

**Figure S2.** Thermogravimetric analysis performed on carbon fiber and lignin (insets) samples under N_2_ atmosphere.

1. **Morphology of fracture surfaces**





CP

CP


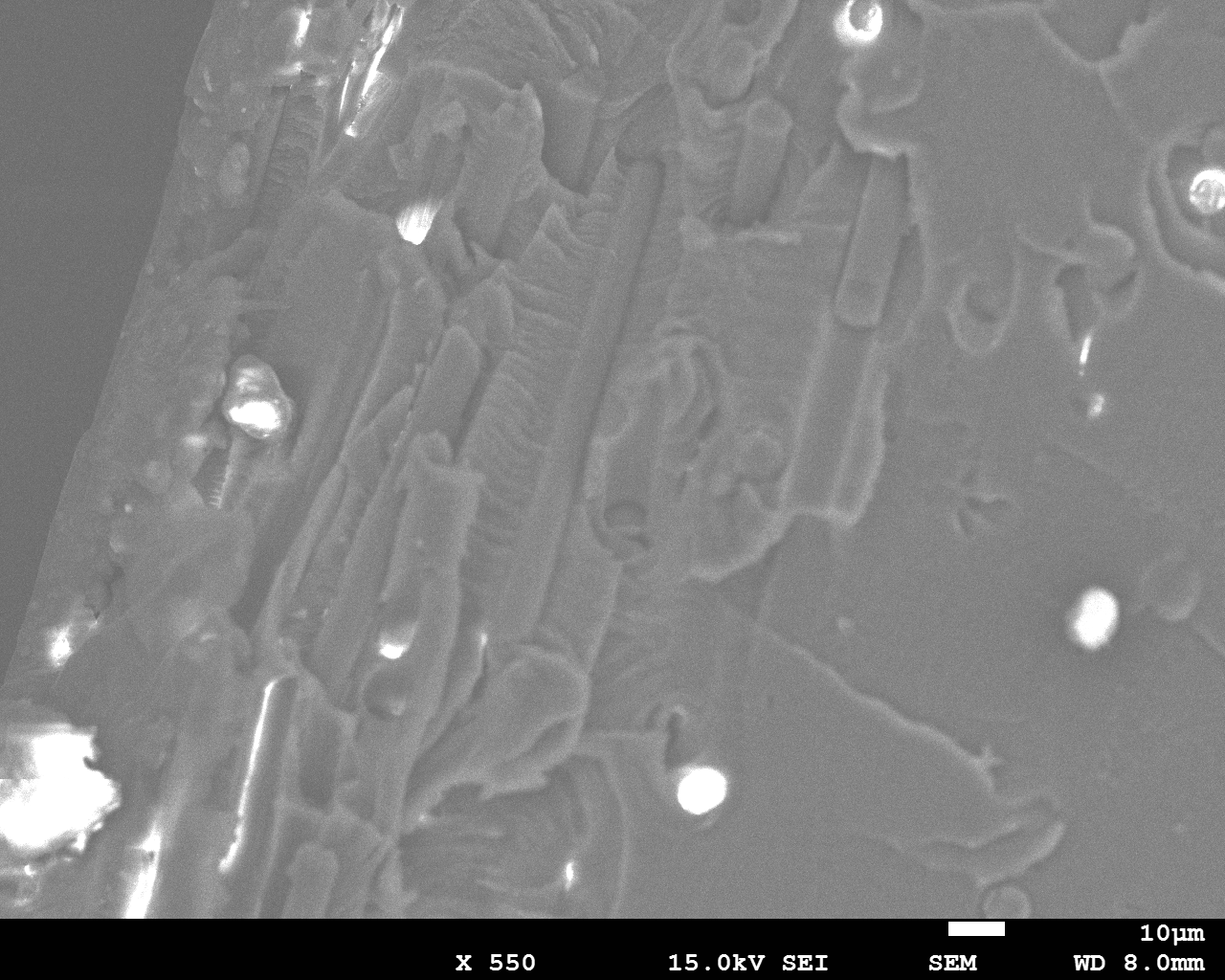

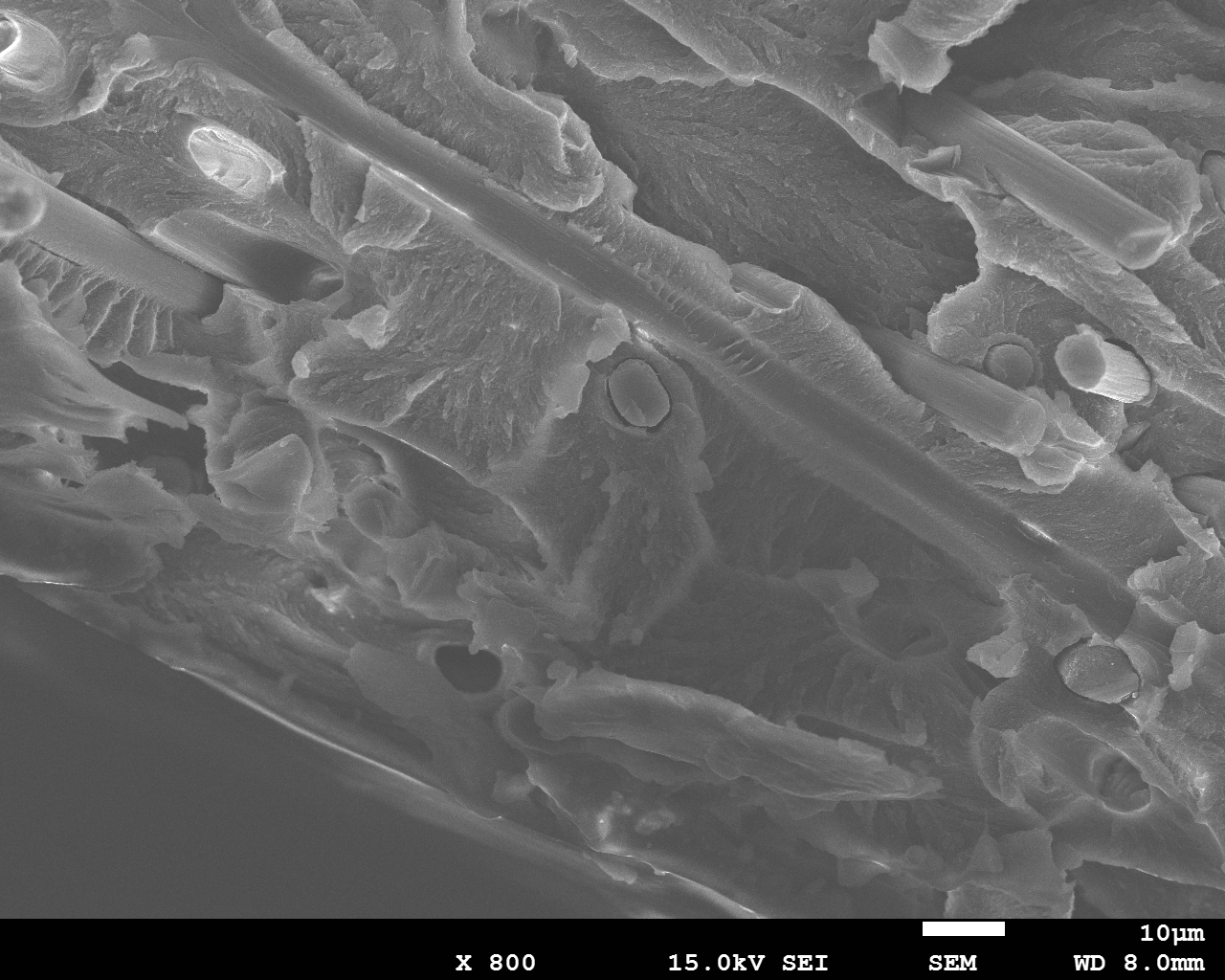

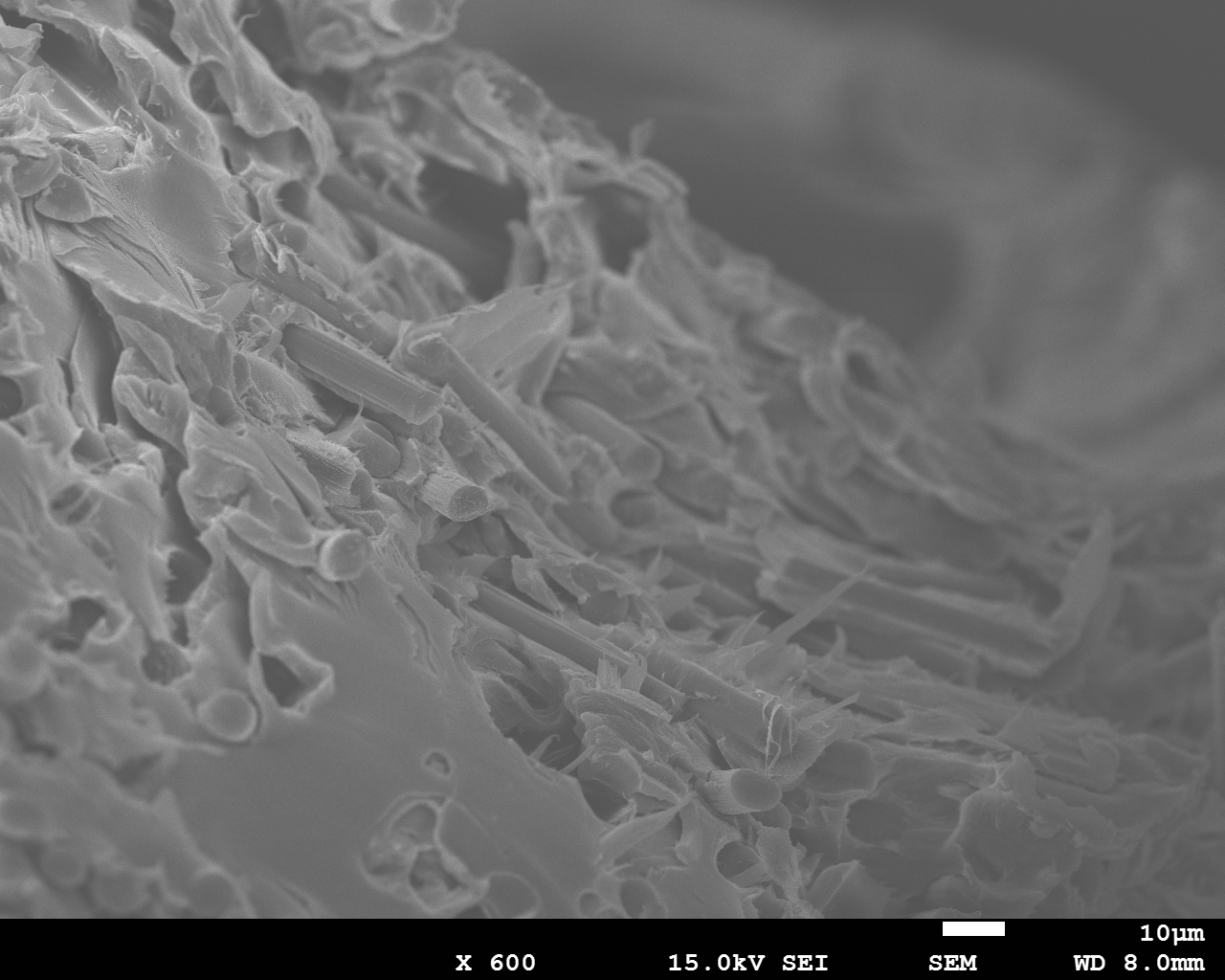

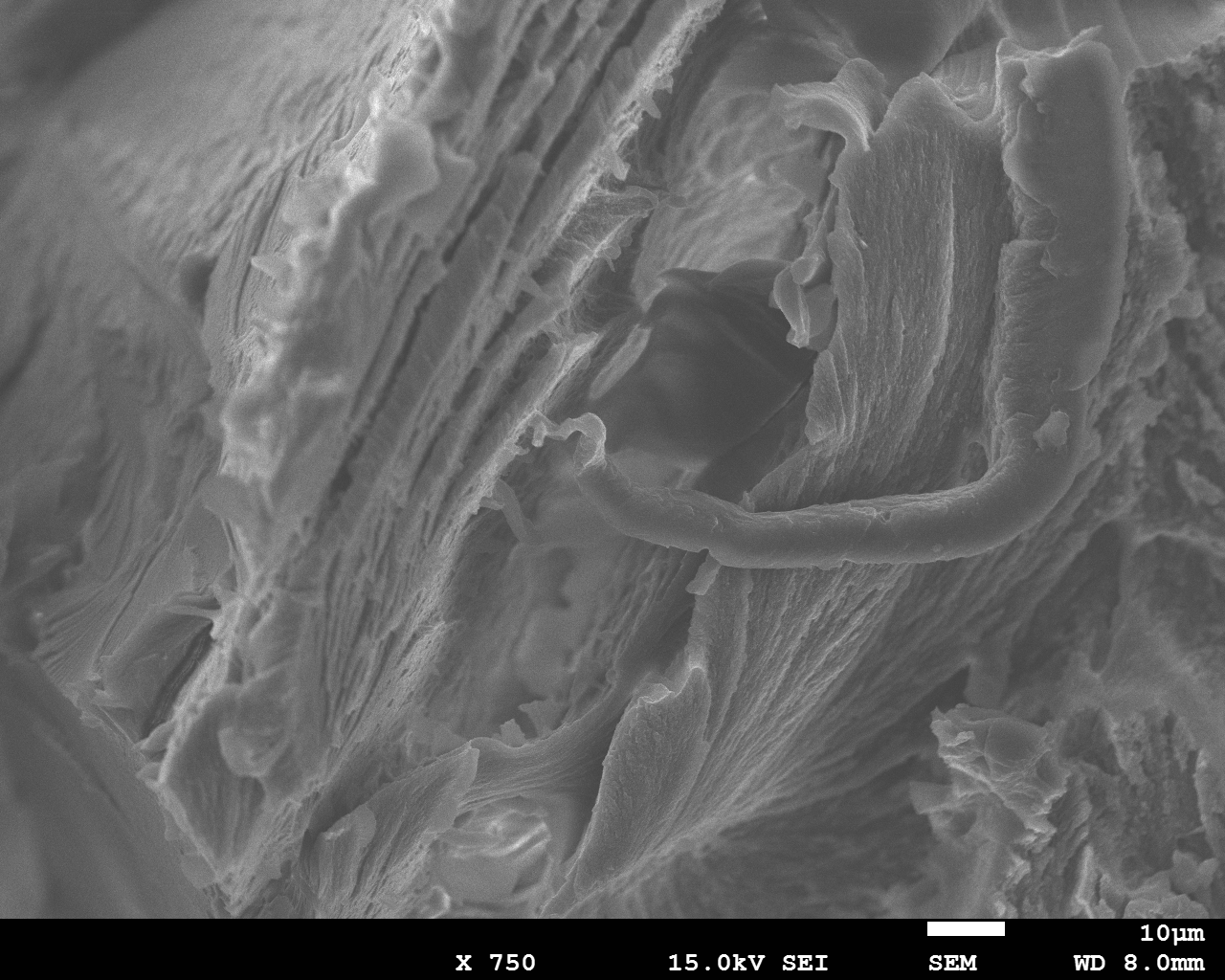





CP + 5% CF (skin)

CP + 5% CF (core)

CP + 10% CF (skin)

CP + 10% CF (core)


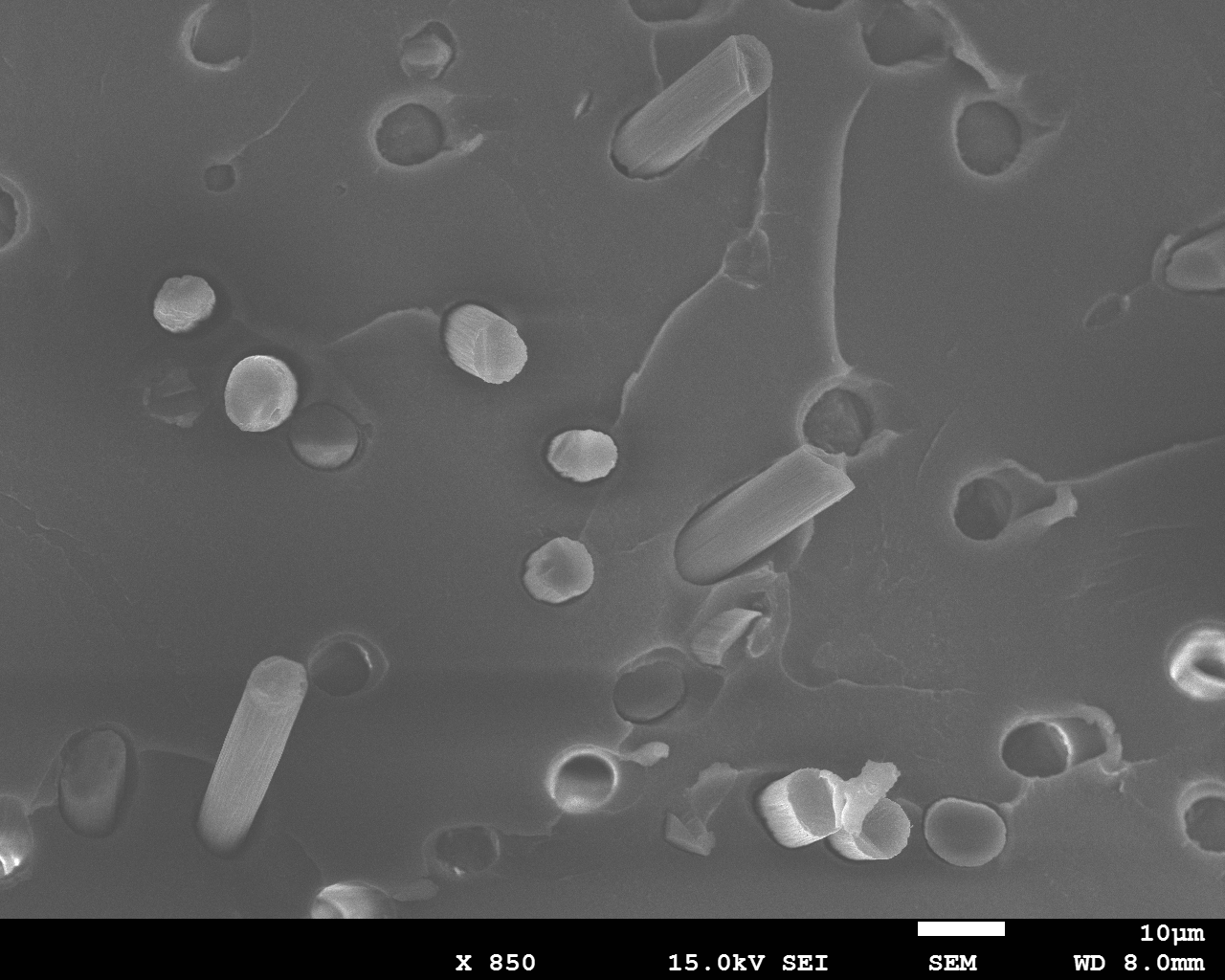

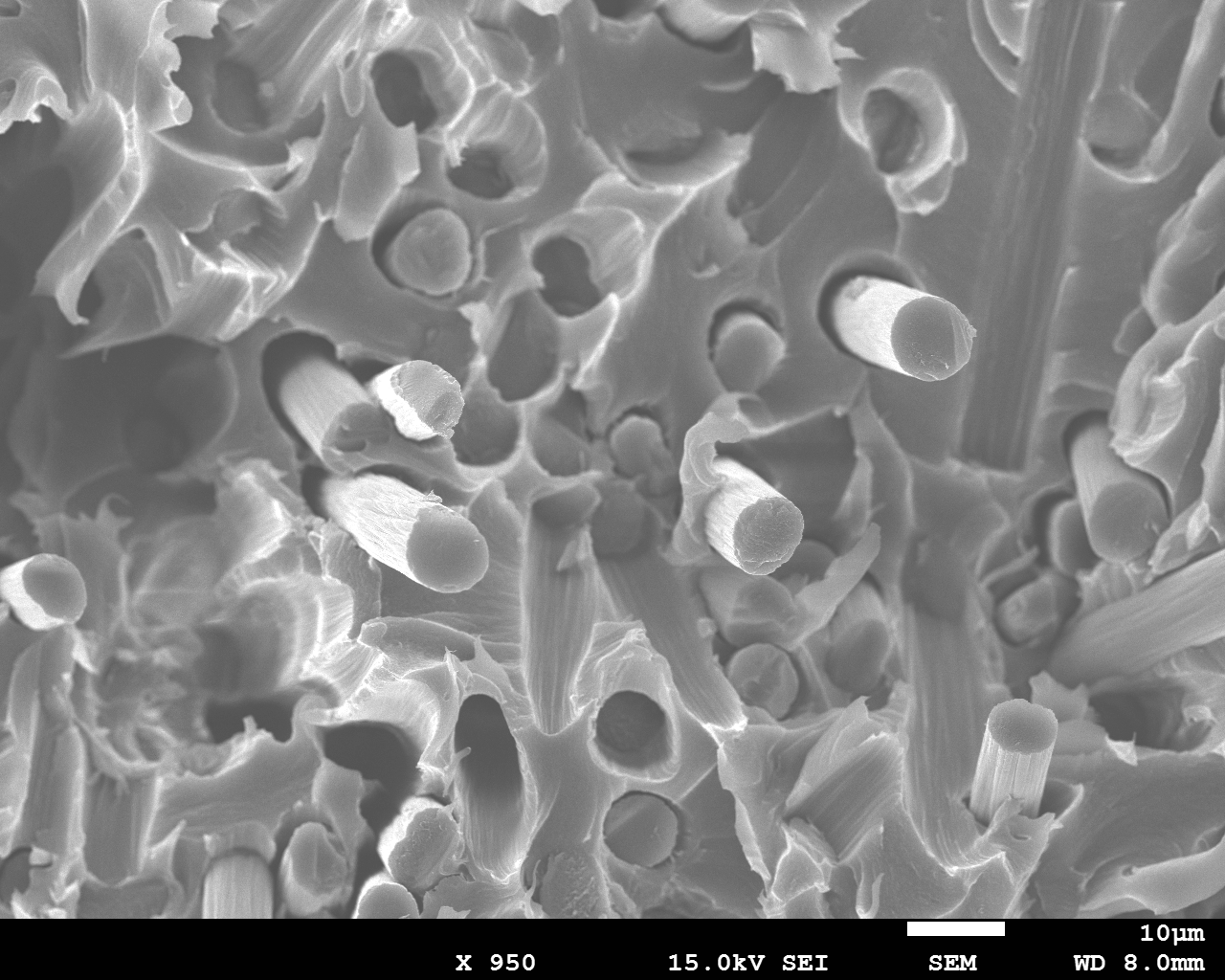


CP + 20% CF (skin)

CP + 20% CF (core)

**Figure S3.** Fracture surfaces of composite samples showing typical skin and core morphology of dumbbell specimens after the tensile test. The abbreviations are as follows: CP – cellulose propionate; CF- carbon fiber.

CP + 5% MCF (core)

CP + 5% MCF (skin)


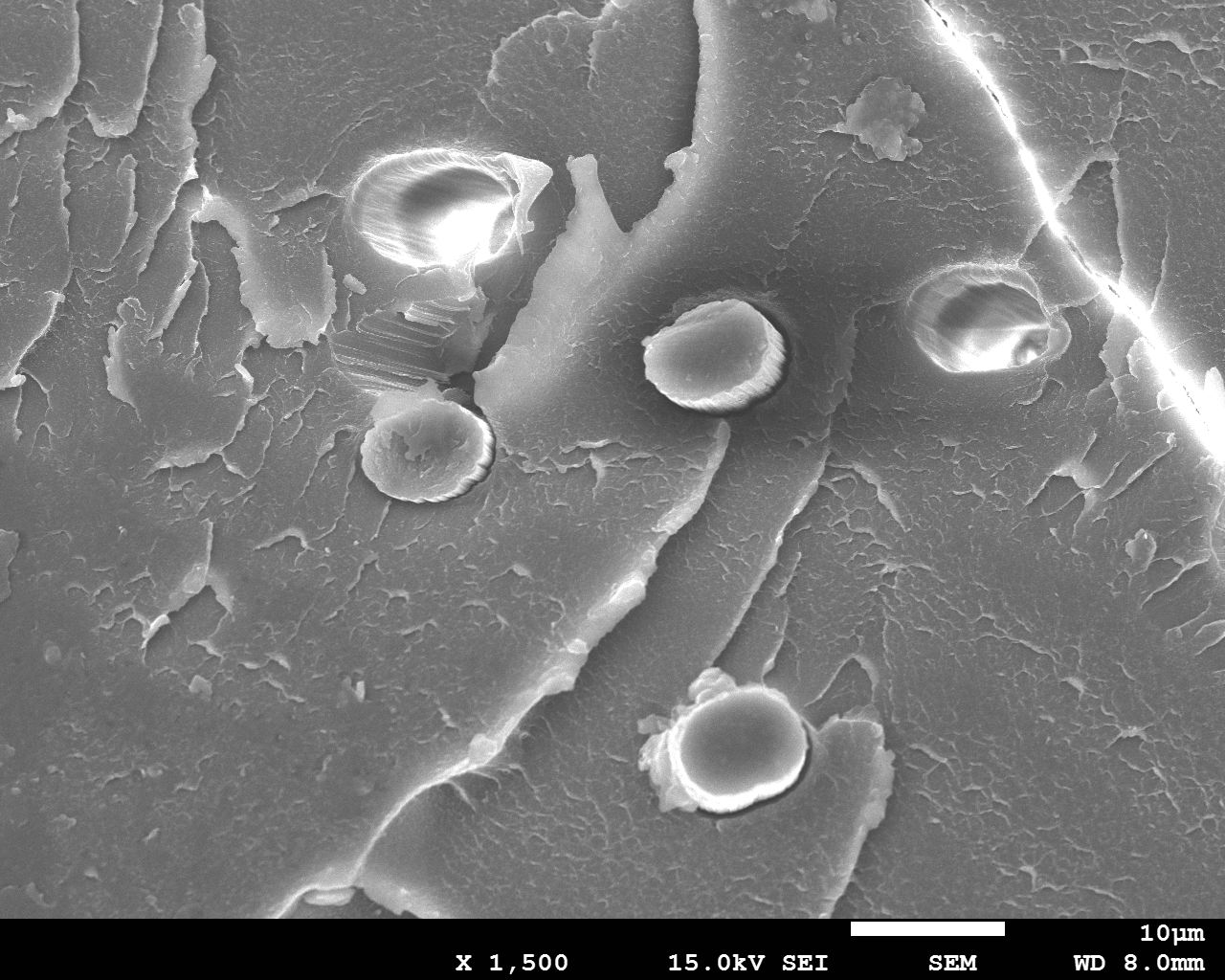

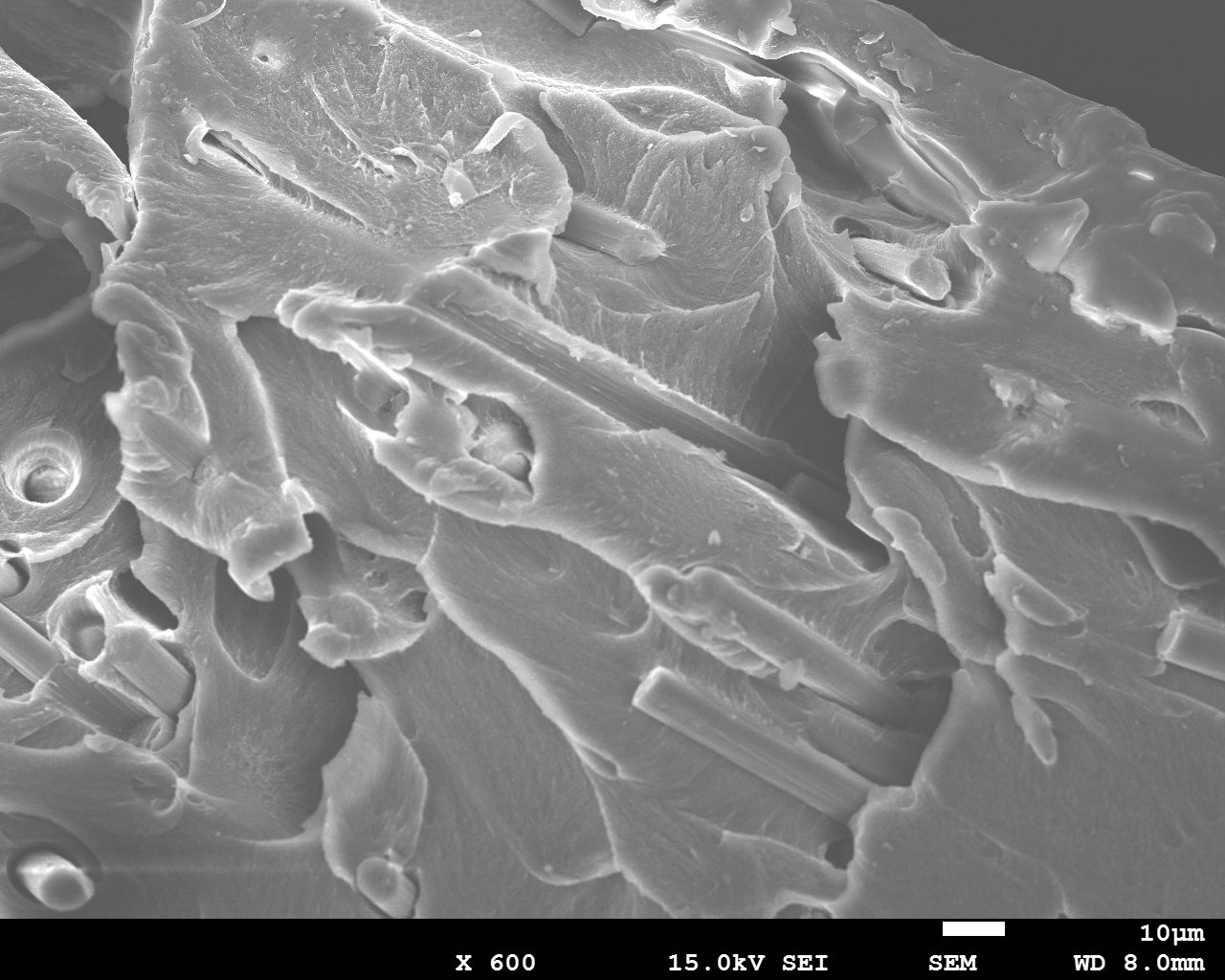







CP + 10% MCF (core)

CP + 10% MCF (skin)

CP + 20% MCF (core)

CP + 20% MCF (skin)









**Figure S4.** Fracture surfaces of composite samples showing typical skin and core morphology of dumbbell specimens after the tensile test. The abbreviations are as follows: CP – cellulose propionate; MCF- modified carbon fiber (lignin coating).







CAB

CAB







CAB + 5% CF (core)

CAB + 5% CF (skin)

CAB + 10% CF (core)

CAB + 10% CF (skin)


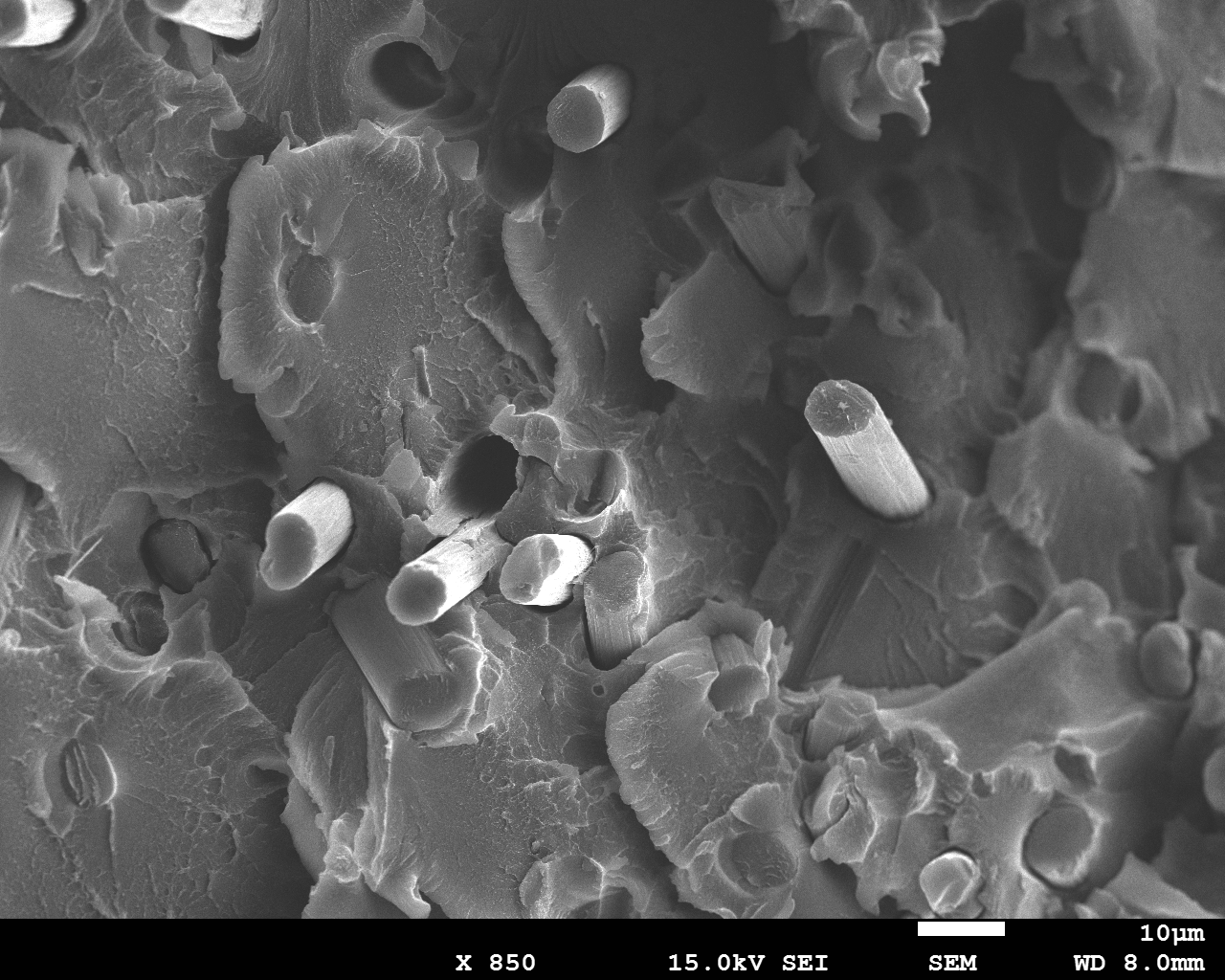

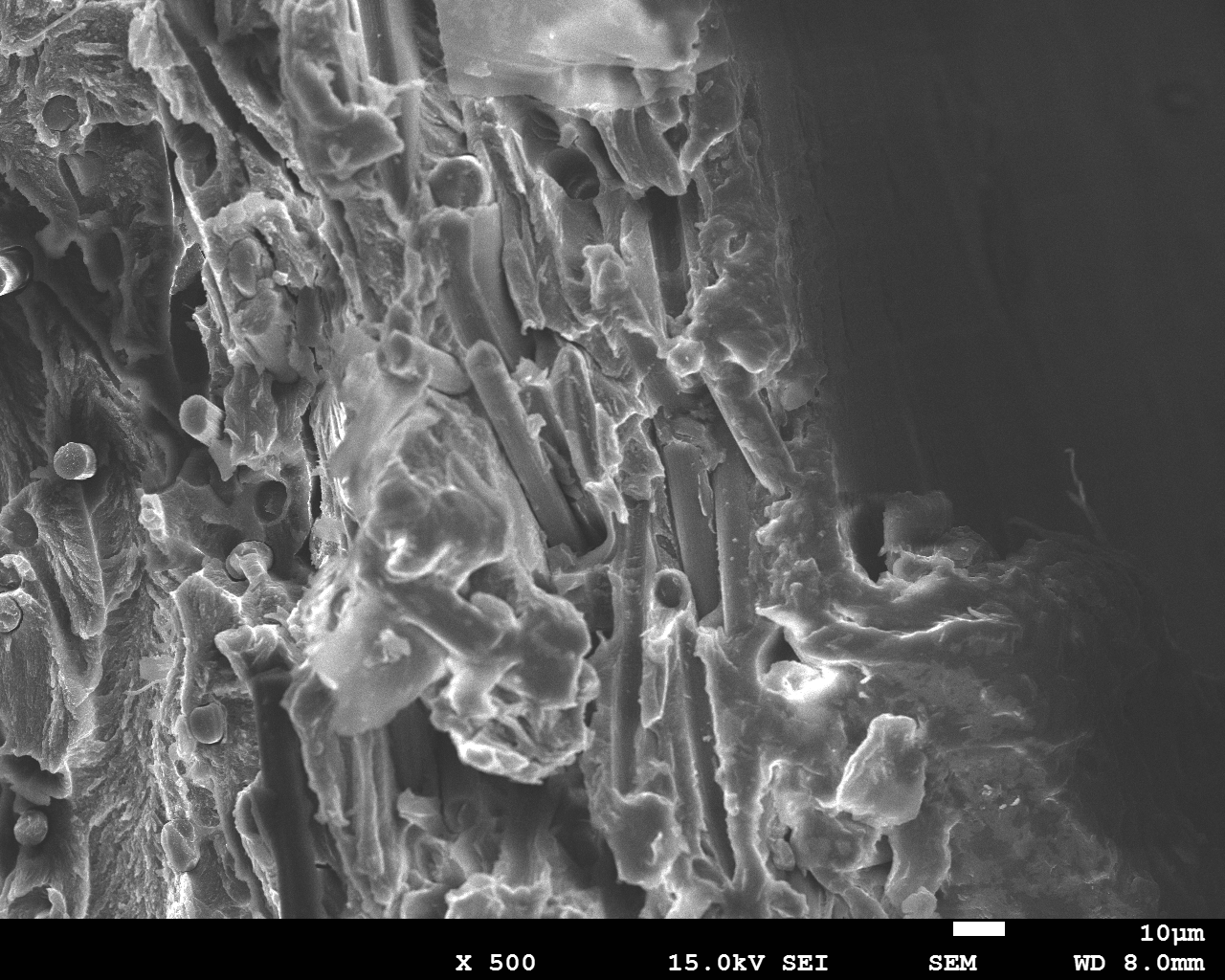


CAB + 20% CF (core)

CAB + 20% CF (skin)





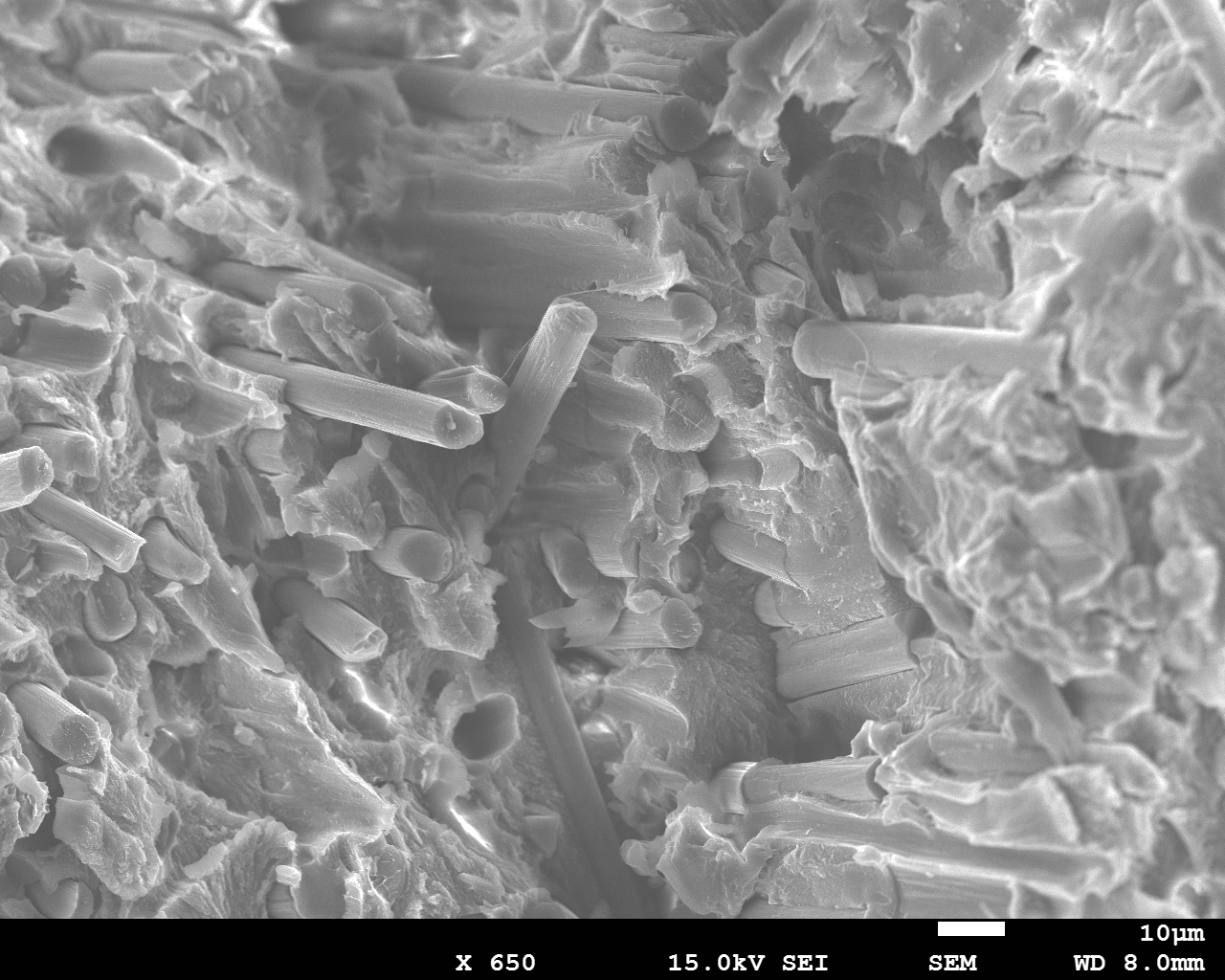


**Figure S5.** Fracture surfaces of composite samples showing typical skin and core morphology of dumbbell specimens after the tensile test. The abbreviations are as follows: CAB – cellulose acetate butyrate; CF- carbon fiber.





CAB + 5% MCF (skin)

CAB + 5% MCF (core)


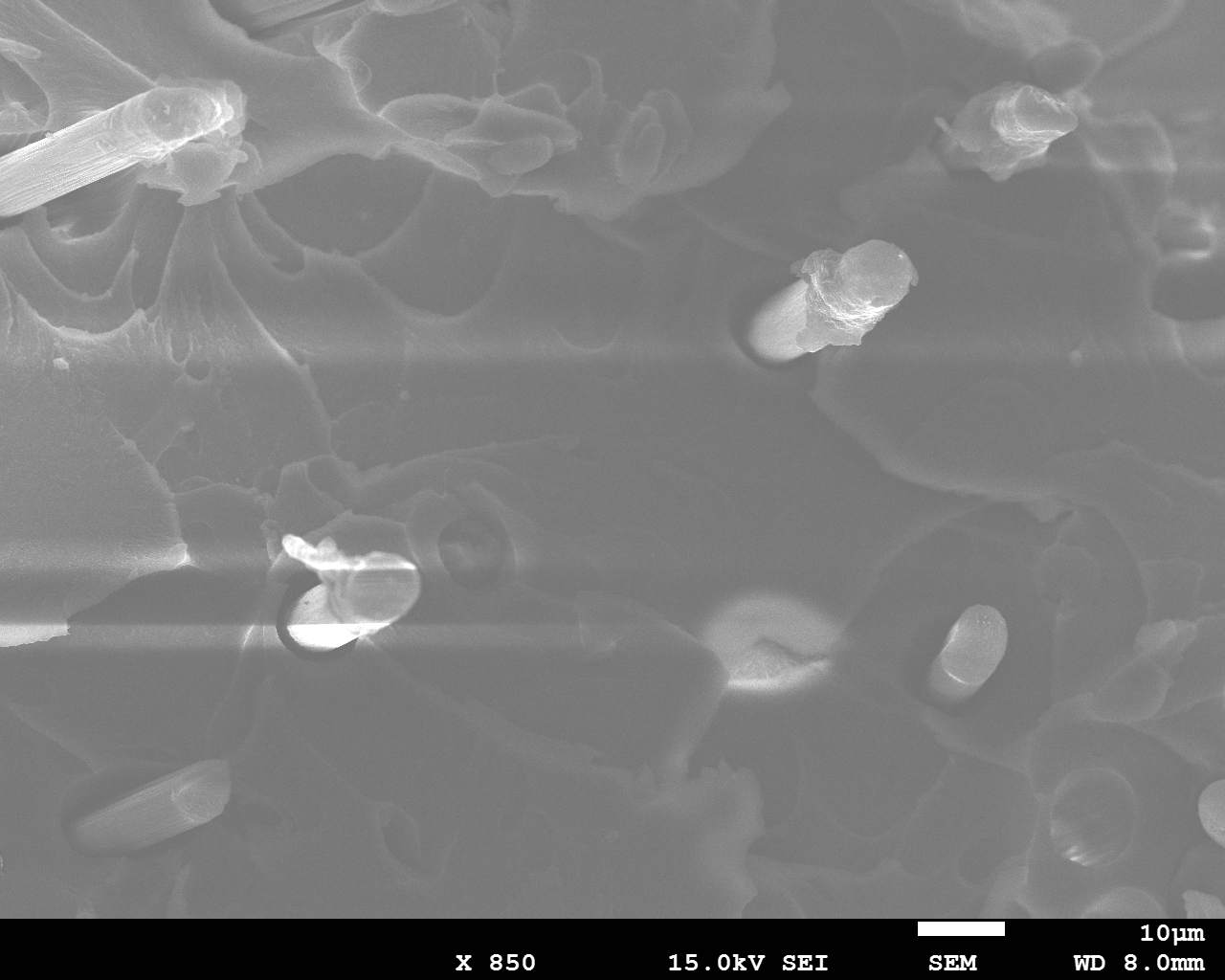

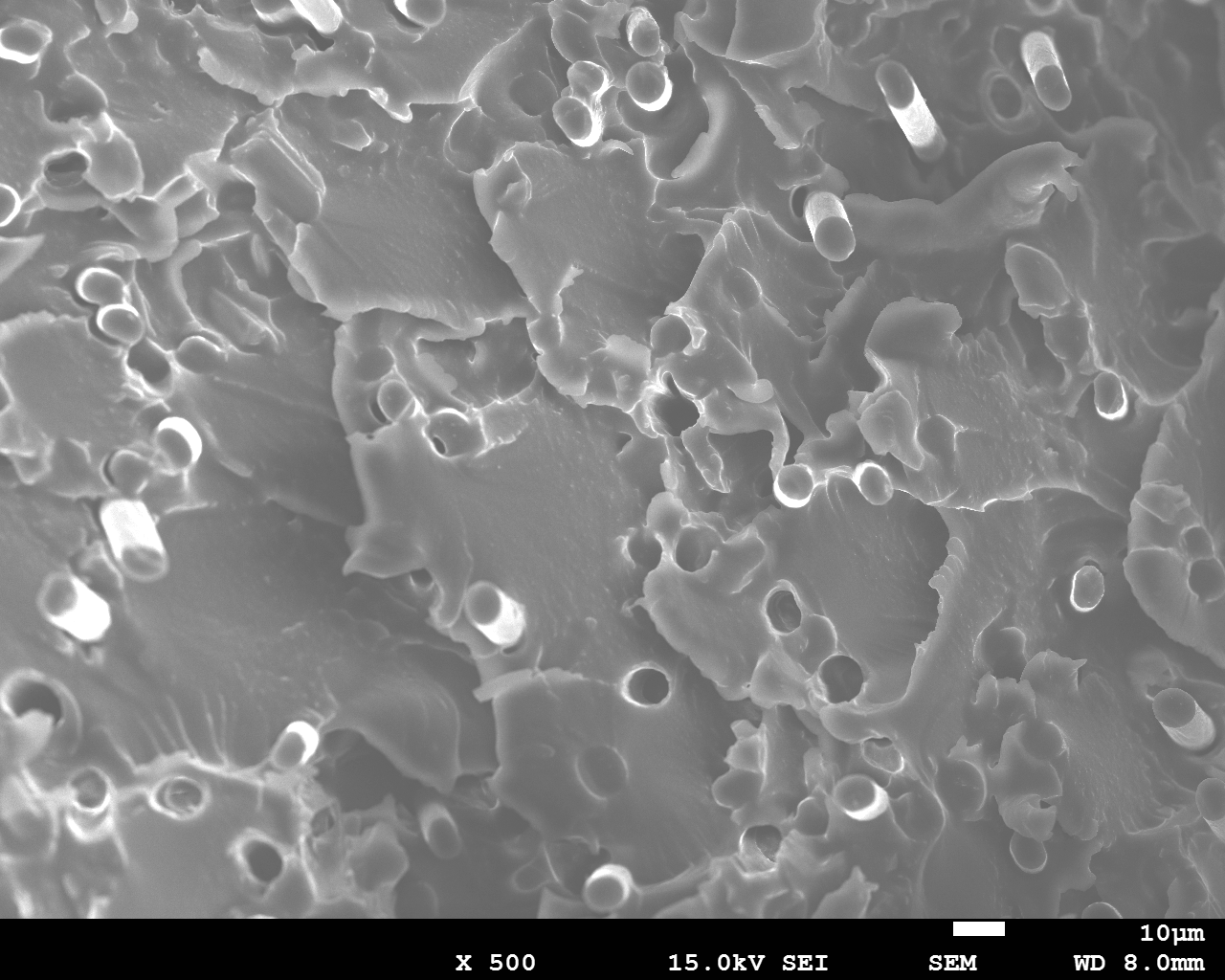


CAB + 10% MCF (core)

CAB + 10% MCF (skin)


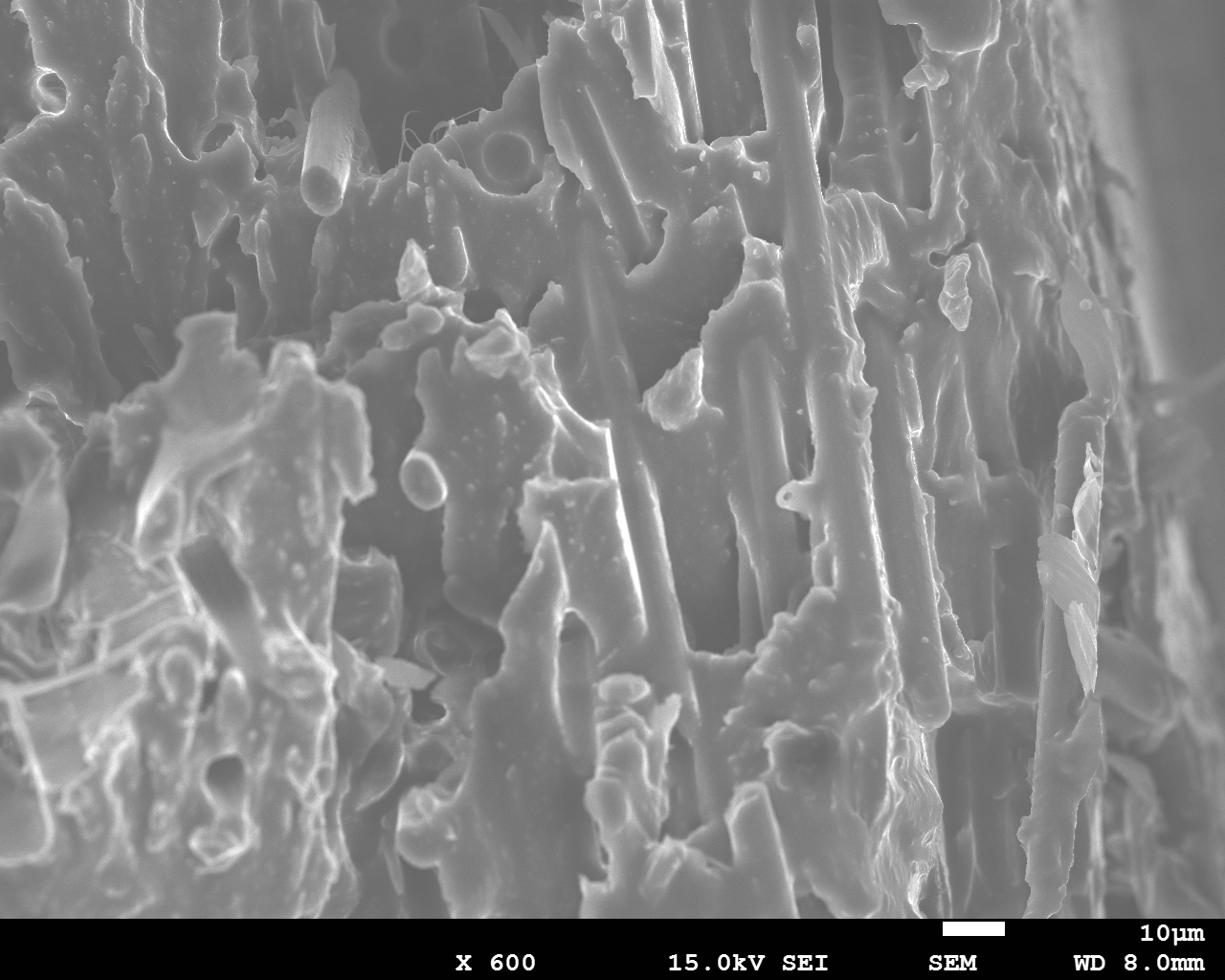

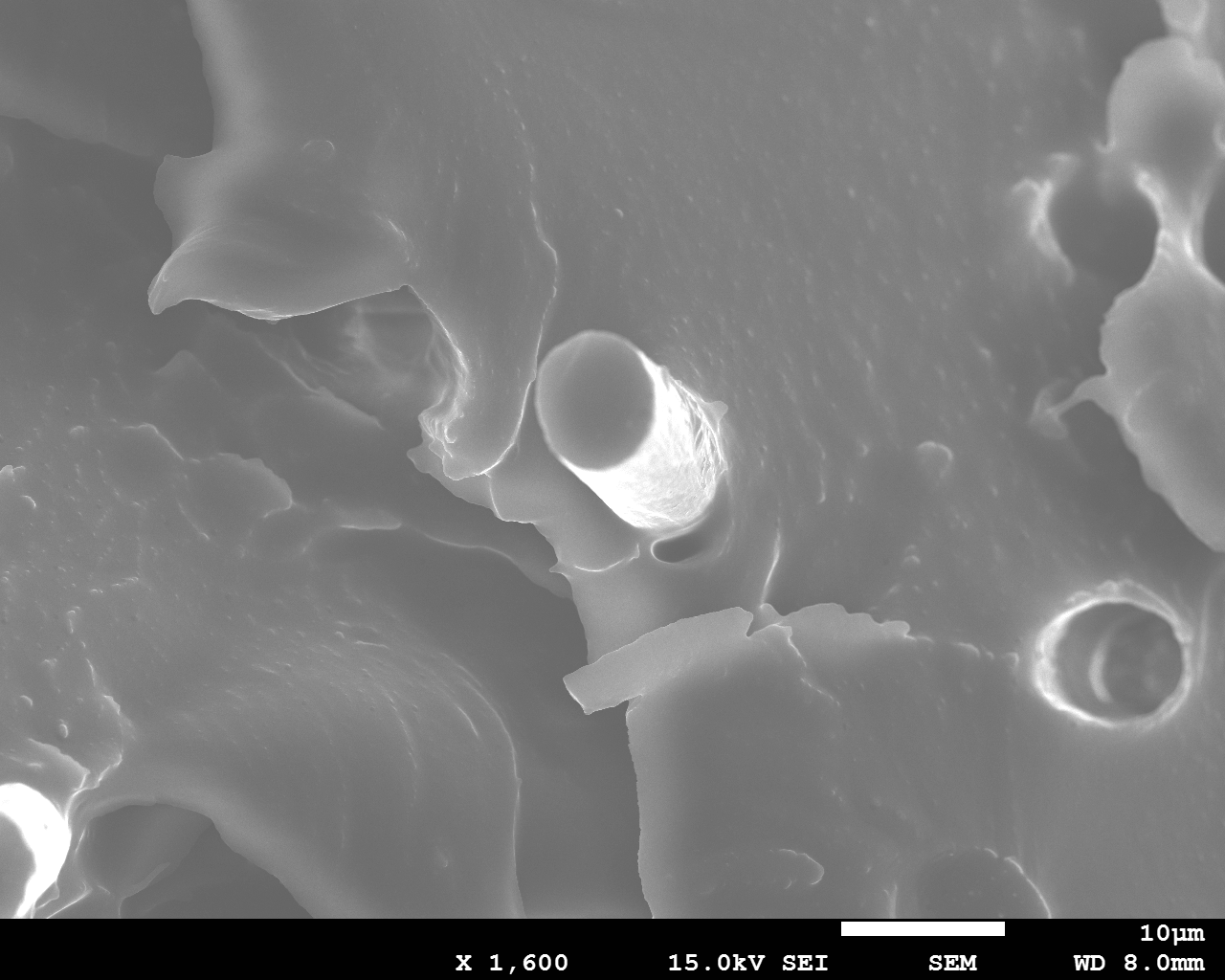




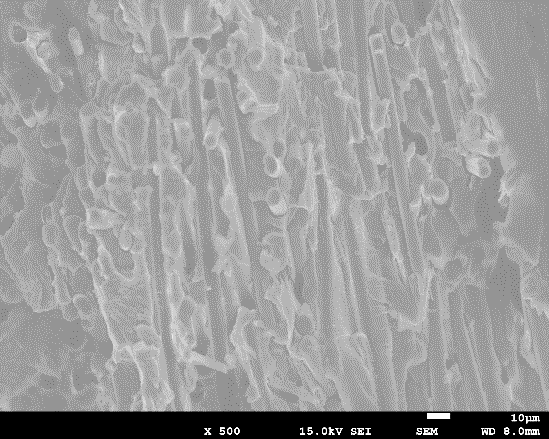


CAB + 20% MCF (core)

CAB + 20% MCF (skin)

**Figure S6.** Fracture surfaces of composite samples showing typical skin and core morphology of dumbbell specimens after the tensile test. The abbreviations are as follows: CAB – cellulose acetate butyrate; MCF- modified carbon fiber (lignin coating).







Nylon 6

Nylon 6







Nylon 6 + 5% CF (core)

Nylon 6 + 5% CF (skin)







Nylon 6 + 10% CF (core)

Nylon 6 + 10% CF (skin)







Nylon 6 + 20% CF (core)

Nylon 6 + 20% CF (skin)

**Figure S7.** Fracture surfaces of composite samples showing typical skin and core morphology of dumbbell specimens after the tensile test. The abbreviations are as follows: CF - carbon fiber.















Nylon 6 + 5% MCF (skin)

Nylon 6 + 5% MCF (core)

Nylon 6 + 10% MCF (core)

Nylon 6 + 10% MCF (skin)

Nylon 6 + 20% MCF (core)

Nylon 6 + 20% MCF (skin)

**Figure S8.** Fracture surfaces of composite samples showing typical skin and core morphology of dumbbell specimens after the tensile test. The abbreviations are as follows: MCF – modified carbon fiber (lignin coating).







PP

PP







PP + 5% CF

PP + 5% CF







PP + 10% CF

PP + 10% CF







PP + 20% CF

PP + 20% CF

**Figure S9.** Fracture surfaces of composite samples after the tensile test. The abbreviations are as follows: PP – polypropylene, CF – carbon fiber. Please note that in case of composites prepared with polypropylene distinguishing skin-core structure is difficult due to the ductile nature of the polymer.









PP + 5% MCF

PP + 5% MCF





PP + 10% MCF

PP + 10% MCF







PP + 20% MCF

PP + 20% MCF

**Figure S10.** Fracture surfaces of composite samples after the tensile test. The abbreviations are as follows: PP – polypropylene, MCF – modified carbon fiber (lignin coating). Please note that in case of composites prepared with polypropylene distinguishing skin-core structure is difficult due to the ductile nature of the polymer.

1. **Fiber length distribution**

**Figure S11.** Fiber length distribution of cellulose propionate based composite samples containing (A) 5 wt%, (B) 10 wt% and (C) 20 wt% unmodified, and (D) 5 wt%, (E) 10 wt% and (F) 20 wt% functionalized carbon fibers.

**Figure S12.** Fiber length distribution of cellulose acetate butyrate based composite samples containing (A) 5 wt%, (B) 10 wt% and (C) 20 wt% unmodified, and (D) 5 wt%, (E) 10 wt% and (F) 20 wt% functionalized carbon fibers.

**Figure S13.** Fiber length distribution of polyamide 6 based composite samples containing (A) 5 wt%, (B) 10 wt% and (C) 20 wt% unmodified, and (D) 5 wt%, (E) 10 wt% and (F) 20 wt% functionalized carbon fibers.

**Figure S14.** Fiber length distribution of polypropylene based composite samples containing (A) 5 wt%, (B) 10 wt% and (C) 20 wt% unmodified, and (D) 5 wt%, (E) 10 wt% and (F) 20 wt% functionalized carbon fibers.
